# Ceramides: A Biologically Attractive Lipid and Advances in Acquisition Strategies

**DOI:** 10.3390/antiox15091050

**Published:** 2026-08-22

**Authors:** Yu Yan, Xinghua Ren, Yue Wu, Li Li, Huanxiang Yuan

**Affiliations:** Beijing Key Lab of Plant Resource Research and Development, Beijing Technology and Business University, Fucheng Road, Haidian District, Beijing 100048, China

**Keywords:** ceramides, redox homeostasis, microbial fermentation, natural extraction, chemical synthesis

## Abstract

Ceramides (Cer), as bioactive substances, hold significant research importance in fields such as biology, medicine, and daily chemicals. As a significant bioactive substance, Cer has garnered considerable attention in various fields in recent years. Especially in the field of daily chemicals, Cer serves as a core functional ingredient in skincare products, which ameliorate skin dryness and sensitivity and reinforce the barrier function of the stratum corneum. Among them, ultra-long-chain Cer assemble compact and ordered epidermal lipid layers to block exogenous oxidative stress, reduce intracellular ROS accumulation, and mitigate lipid-peroxidation-mediated skin barrier damage, while excess medium- and short-chain Cer disrupt the ordered arrangement of stratum corneum lipids and further aggravate cutaneous oxidative-stress imbalance. Such chain-length-dependent functional divergence reflects the core regulatory effect of structure–activity relationships on cutaneous physiological phenotypes. The acquisition of Cer is of great significance for advancing their research and applications. Currently, there is a lack of comprehensive reviews that introduce the acquisition pathways of Cer and discuss efficient preparation strategies for different methods. Cer and their related raw materials can be obtained through three main pathways: microbial fermentation, natural extraction, and chemical synthesis. This review provides a comprehensive comparison and analysis of these three approaches, focusing on recent advancements and developments in the field. The purpose of this review is to enable researchers to gain a comprehensive understanding of the current research status of the preparation strategies for biological Cer. And the discussion of these studies will be propitious to the future improvement of green synthesis and large-scale production, further promoting the wide application of Cer.

## 1. Introduction

Ceramides (Cer), as bioactive sphingolipid derivatives, serve as pivotal macromolecules in cellular systems, exerting pleiotropic functions spanning the structural maintenance of membrane architecture to the modulation of signaling pathways. Structurally comprising long-chain base (LCB) amide-linked fatty acid (FA) moieties [1], their physiological properties are principally governed by FA characteristics, including chain length and saturation degree. Based on acyl chain length, Cer are typically classified into four categories: medium- (C12–C20), long- (C20–C24), very long- (C24–C26), and ultra-long-chain (≥C26) Cer. These sphingolipids demonstrate multidimensional bioactivities across biological disciplines. Biologically, they traverse the blood–brain barrier as signaling mediators in apoptosis and proliferation pathways. Medically, they demonstrate therapeutic potential in antitumor and antiviral applications. Dermatologically, they are critical for maintaining epidermal homeostasis through regulating skin hydration and facilitating barrier repair processes.

### 1.1. Biological Aspects

Cer constitute a family of key bioactive sphingolipid molecules extensively involved in multiple core human biological processes. Owing to their unique structural properties and subtype heterogeneity, Cer exert indispensable functions in transmembrane substance transport, cellular signal modulation, cell proliferation and apoptosis, and systemic redox balance regulation, acting as essential functional molecules for maintaining physiological homeostasis. The blood–brain barrier consists of brain capillary endothelial cells, tight junctions, basement membranes and astrocytic endfeet [2]. Cumulative in vitro model studies have validated that plant-derived Cer possesses favorable blood–brain-barrier-penetrating capacity [3]. Upon oral administration, their precursor glucosylceramides (GlcCer) are hydrolyzed by gastrointestinal digestive enzymes to generate free Cer and glucose [4,5]. Sphingolipid metabolic remodeling takes place during trans-barrier transport, whereby Cer participate in central physiological regulation. As pivotal intracellular messengers, ceramide derivatives mediate cell-to-cell communication and immune reprogramming [6,7]. Sphingolipidomic data reveal that the expression signature of ceramide-1-phosphate (C1P) can directly govern the migratory phenotype of macrophages [8,9,10]. Down-regulated C1P in M1-type macrophages impedes cytoskeleton remodeling and impairs migratory potential, triggering cellular retention and ultimately disturbing the dynamic course of inflammatory responses. Sphingolipid metabolic profiles of Cer markedly modulate neuronal proliferative activity and are strongly linked to the pathological progression of mood disorders. Sustained neurogenesis occurs within the adult hippocampus, and impairment of this process serves as a major predisposing factor for depression [11,12]. The acid sphingomyelinase-dependent biosynthetic pathway of Cer represents a central therapeutic target for antidepressants [13]. Further evidence from gene-knockout animal models confirms that endothelium-derived C16-type Cer robustly suppresses neuronal proliferation, implying that dysregulated sphingolipid metabolism acts as a novel molecular mechanism governing neurogenesis [14]. Cer also orchestrates apoptotic cascades via the mitochondrial pathway [15]. Voltage-dependent anion channel 2 (VDAC2) residing on the outer mitochondrial membrane specifically recognizes and binds ceramide molecules, serving as a core effector protein for ceramide-provoked apoptosis [16]. Molecular dynamics simulation results demonstrate that VDAC2 deletion substantially abrogates ceramide-driven mitochondrial apoptosis, elucidating the targeted regulatory mechanism of this signaling axis [17]. Notably, Cer exerts carbon-chain-dependent bidirectional regulatory effects on cellular redox homeostasis. Short- and medium-chain Cer (C16:0, C18:0) exacerbate cellular oxidative injury by disrupting the mitochondrial electron-transport chain, boosting ROS accumulation and inducing mitochondrial fragmentation [18,19,20,21]. In contrast, ultra-long-chain Cer (C22–C24) catalyzed by CerS2 antagonize pore formation triggered by C16:0-Cer on the outer mitochondrial membrane, stabilize membrane permeability, reduce ROS release, and preserves mitochondrial redox homeostasis. These two subtypes of Cer exert functionally antagonistic effects in mitochondrial respiration and membrane homeostasis, collectively forming the molecular basis whereby sphingolipids modulate cellular oxidative balance [22].

### 1.2. Medical Aspects

As core bioactive molecules in sphingolipid metabolism, Cer exhibit considerable clinical-translation potential in tumors, viral infections, cardiovascular disorders and neurodegenerative diseases via multi-layered regulation of apoptosis, viral replication, and the oxidative-inflammatory network. In anti-tumor research, Cer mediate the balance between proliferation and death of tumor cells and serve as a vital lipid target for tumor intervention [23,24]. Existing review has illustrated their regulatory significance in breast cancer therapy [25]. Neoadjuvant strategies, including endocrine therapy, chemotherapy, and radiotherapy, can trigger the accumulation of Cer in tumor cells and further induce tumor cell apoptosis; although distinct therapeutic approaches elicit Cer buildup through disparate molecular mechanisms, Cer-dependent cell death represents a shared feature underlying the efficacy of multiple anti-tumor regimens [26,27]. During viral infection, Cer interfere with viral replication and latency cycles by remodeling host sphingolipid metabolism [28,29,30]. Influenza A virus infection elevates levels of Cer in pulmonary epithelial cells in a time- and dose-dependent manner, and exogenous ceramide administration activates the de novo synthesis pathway to markedly suppress viral replication and confer cytoprotection [31]. By contrast, Epstein–Barr virus (EBV) maintains its latent state and evades host clearance by reducing intracellular content of Cer, whereas increased Cer abundance activates the ERK1/2-CREB pathway, upregulates immediate-early genes, and drives viruses into the lytic cycle for EBV elimination, which reflects the bidirectional regulatory effects of Cer upon viral infection [32]. For cardiovascular diseases, Cer isoform with different acyl-chain lengths influence disease prognosis and metabolic homeostasis [33,34]. The plasma C24:0/C16:0-Cer ratio acts as an independent prognostic biomarker for heart failure with preserved ejection fraction: C16:0-Cer exert pro-inflammatory and cytotoxic effects while C24:0-Cer confer protective functions, and a decreased ratio predicts elevated risks of cardiovascular mortality and heart-failure-related hospitalization [35]. Under obesity and type 2 diabetes, the specific reduction of C18:0-Cer within mitochondrial–endoplasmic–reticulum contact sites of skeletal muscle is strongly correlated with improved insulin sensitivity, indicating that subcellular-localized Cer participates in the modulation of metabolism-associated cardiovascular injury [36].

### 1.3. Skin Physiological Roles of Ceramides

Cer are core lipid components that maintain cutaneous physiological homeostasis. Owing to structural features and biological activities differentiated by acyl-chain length, Cer exert central regulatory roles in skin-barrier formation, keratinocyte adhesion and consolidation, as well as the modulation of cutaneous oxidative-stress homeostasis, and represent pivotal functional lipids for preserving normal epidermal physiological functions. As the main structural constituent of the skin barrier, the stratum corneum is composed of keratinocytes and intercellular lipids, among which Cer, free fatty acids, and cholesterol jointly construct the typical “brick-and-mortar” architecture that effectively blocks the invasion of exogenous harmful substances and safeguards epidermal-barrier integrity [37,38]. During epidermal keratinization and differentiation, newly synthesized Cer are metabolized into GlcCer and sphingomyelins and stored within lamellar bodies; after being transported to the interface between the granular layer and the stratum corneum, these lipids are reconverted into functional Cer under the catalysis of β-glucocerebrosidase and sphingomyelinase, thus accomplishing the assembly and repair of the skin-barrier structure [39]. Atopic dermatitis is pathologically characterized by impaired skin barrier, accompanied by marked abnormalities in the content and species of stratum corneum Cer, and such dysregulation stems from imbalanced expression, catalytic activity, and spatial localization of enzymes involved in lipid synthesis and degradation [40,41]. As the key rate-limiting enzyme for epidermal production of Cer, β-glucocerebrosidase catalyzes the hydrolysis of GlcCer to yield functional Cer [42], and experimental evidence has demonstrated that the activity of this enzyme is substantially reduced in the epidermis of atopic dermatitis patients, which directly triggers cutaneous lipid metabolic disturbance and barrier insufficiency [43]. Besides cross-linking with cholesterol and fatty acids to form compact lamellar bilayers, Cer also builds a moisturizing network in the stratum corneum by binding free water molecules to stabilize skin hydration status, which is essential for barrier-structure stabilization and moisturization maintenance [44,45,46]; furthermore, Cer reinforce intercellular structural stability via covalent cross-linking mechanisms [47]. A highly cross-linked cornified envelope lies on the surface of keratinocytes, where specific lipids such as ω-hydroxyCer can covalently conjugate with glutamate residues of envelope-associated functional proteins, generating a stable monolayer cornified lipid envelope and greatly enhancing overall epidermal compactness [48]. Reduced stratum-corneum ceramide levels markedly impair intercellular adhesion, directly resulting in skin dryness, desquamation, and scaling; ceramide content shows a significant correlation with the severity of skin dryness, whereas the exogenous supplementation of Cer effectively strengthens keratinocyte adhesion and alleviates diverse dry-skin conditions [49,50]. More importantly, Cer modulate cutaneous oxidative stress in an environment-dependent and structure-specific manner, with distinct functional outcomes among different acyl-chain subtypes. Endogenously enriched ultra-long-chain Cer in the epidermis assembles highly ordered compact lipid layers to physically block exogenous oxidative stimuli, including ultraviolet radiation and air pollutants, and lower basal cutaneous oxidative burden [51]. Oxidative stress and ultraviolet radiation can cleave acyl chains of Cer, leading to the depletion of protective ultra-long-chain fractions and aberrant accumulation of damaging short-chain Cer, which disrupt ordered lipid arrangement, increase membrane permeability, and trigger barrier breakdown. Under barrier-damaged and photo-damaged conditions, pathological accumulation of medium- and short-chain Cer, such as C16-Cer, further amplifies oxidative stress and inflammatory responses, induces aberrant keratinocyte apoptosis, and accelerates skin photoaging, pigmentation, and fine-line formation [52]. Accordingly, ultra-long-chain Cer can stabilize cutaneous lipid architecture and counteract oxidative injury, which provides important theoretical foundations for skin barrier repair and antioxidant skincare applications.

## 2. Types and Sources of Ceramides

Cer are compounds formed through the acylation reaction between LCB and FA. There are mainly twelve types of Cer in the human body, classified as shown in Table 1, which are formed by the combination of three FAs and four LCBs [53].

The primary methods for obtaining Cer include microbial fermentation, natural extraction, and chemical synthesis. Microbial fermentation has become a commonly used method in recent years for preparing Cer precursor compounds. This involves fermenting microorganisms such as *Pichia ciferri* (*P. ciferri*), *Wickerhamomyces ciferrii* (*W. ciferrii*), and *Saccharomyces cerevisiae* (*S. cerevisiae*) under specific conditions to produce tetraacetylphytosphingosine (TAPS), which is then deacetylated to yield phytosphingosine (Phyto-Sph). The Phyto-Sph is subsequently combined with different FA to synthesize the desired Cer [54]. However, the types of LCB obtained through microbial fermentation are limited, primarily yielding Phyto-Sph, and thus only Phyto-Sph-related Cer can be produced. Natural extraction of Cer can be sourced from both animals and plants. Cer derived from animal brains poses a risk of disease transmission and is therefore unsuitable for use in cosmetics [55]. Additionally, plant extraction methods are constrained by the growth cycles and seasons of plants, and the extraction yield is relatively low. Chemical synthesis involves a series of chemical reactions to acquire the required FA and various LCB separately, followed by the acylation of the synthesized FA and LCB to produce the desired Cer. Alternatively, the target Cer can be synthesized directly without using FA and LCB as intermediates. Chemical synthesis not only improves the yield of Cer but also allows for the diversity of FA and LCB, offering significant potential for widespread applications across various fields.

## 3. Acquisition of Ceramides

As key functional lipids in the sphingolipid metabolic pathway, Cer exhibit prominent functional divergence among subtypes with different acyl-chain lengths and various origins. Plant-derived Cer additionally possess specific blood–brain-barrier-permeable capacity, and the physiological roles of individual subtypes cannot be mutually substituted. Standardized, high-purity Cer as test substrates are indispensable for investigations covering the pathological mechanisms of central nervous system disorders, targeted pharmacological research on tumors and cardiovascular diseases, as well as experiments concerning skin-barrier physiology and photoaging. Collectively, efficient preparation and acquisition systems for Cer serve as a core prerequisite for systematically elucidating the molecular mechanisms underlying sphingolipid-mediated regulation and promoting the clinical translation and industrialization of central nervous system drugs, clinical targeted agents, and skin-repairing preparations.

### 3.1. Microbial Fermentation

In 1927, Wickerham isolated an unknown yeast from soil, later naming it *W. ciferrii*. In 1954, Wickerham and his colleagues cultured *W. ciferrii* to produce a mating strain, designated NRRL Y-1031. For the first time, they extracted a large quantity of crystals from this strain, identifying a previously unreported compound named TAPS [56]. In the following years, the Wickerham team isolated the F-60-10 strain from the NRRL Y-1031 mating strain, achieving a TAPS yield of 0.485 g/L, marking the first reported TAPS production from *W. ciferrii* [57]. Subsequently, researchers optimized strains and fermentation conditions using various biotechnological approaches, resulting in high-yield fermentation strains. A high-level fermentative growth strain, DSCC 7-25, was isolated from the parental strain NRRL Y-1301. The batch fermentation was conducted using a fed-batch method, with the yeast–malt–glycerol medium selected as the culture medium. The medium composition of the high-yield strain included 0.4% (*w*/*v*) yeast extract, 0.4% (*w*/*v*) malt extract, 0.5% (*w*/*v*) peptone, and an initial glycerol concentration of 16% (*w*/*v*). Glycerol was added gradually during fermentation to achieve a final concentration of 180 g/L. Additionally, 15 mM CaCl_2_, 0.7% (*w*/*v*) sodium citrate, and 0.5% (*w*/*v*) serine were added, as calcium ions and serine synergistically activate serine-palmitoyltransferase activity, significantly enhancing TAPS synthesis. The fermentation parameters were as follows: temperature 25 °C, agitation speed 210 rpm, aeration rate 0.2 vvm, initial pH 7.5, and fermentation duration 108 h. After fermentation, the suspension was transferred to an aging tank and incubated at 3–5 °C for 48 h to promote cell sedimentation and product enrichment. Subsequently, TAPS was purified via organic solvent extraction and silica gel column chromatography, yielding a purity exceeding 95%. Under these conditions, the *P. ciferrii* strain DSCC 7-25 exhibited robust growth, with simultaneous increases in biomass and product yield, achieving a stable TAPS yield of 22.10 g/L, which is the highest yield reported to date for TAPS production via microbial fermentation [58].

#### 3.1.1. Strain Mutagenesis to Enhance Ceramide Precursor Yield

Mutagenesis breeding is a method that utilizes physical and chemical factors to induce genetic variations in organisms, followed by selective breeding to develop new strains. This approach promotes gene mutations during the organism’s life cycle, leading to improved strains. Common physical factors include X-rays and lasers, while chemical factors include alkylating agents and base analogs [59].

Park et al. employed fluorescence-activated cell sorting (FACS) to randomly screen a mutant yeast library to enhance TAPS production [60]. To isolate high-yield TAPS strains from an ethylmethylsulfonate (EMS)-induced mutant library, they established a high-throughput screening platform based on BODIPY and FACS (BODIPY is a class of near-infrared short-wavelength fluorescent dyes which can be used as a tracer dye for TAPS in yeast cells) [61]. After multiple rounds of screening, they selected seven mutant strains (M6, M28, M35, M36, M38, M39, and M40) with the highest yields. Among these, the M40 mutant strain exhibited a 5.23-fold increase in TAPS yield (103.15 mg/L) compared to the wild-type strain (19.79 mg/L). Subsequently, they investigated the effects of different experimental conditions on TAPS production. Regarding the influence of medium pH, batch cultures of the wild-type and M40 mutant strains were conducted using glucose as the carbon source. When the medium pH was below 3.5, both strains showed slow growth. After adding a pH regulator to maintain the pH at 5.4, TAPS production significantly increased. The M40 mutant strain achieved a TAPS yield of 1.495 g/L after 62 h, 1.54 times higher than the wild-type strain (0.973 g/L). Additionally, the TAPS production rate of the mutant strain was 29.9 mg/L/h, 1.6 times higher than that of the wild-type strain (18.7 mg/L/h). These results suggested that TAPS yield could be further improved. Therefore, they explored the effects of different carbon sources on fermentation. Flask cultures of the wild-type and M40 strains were conducted in TAPS medium supplemented with glucose or molasses, maintaining the pH at 5.4. When glucose was used as the carbon source, TAPS production was 97.31 ± 4.24 mg/L, whereas molasses significantly increased TAPS yield to 176.43 ± 5.49 mg/L. Next, larger-scale batch cultures were performed. Unlike flask cultures, yeast exhibited slow growth initially when molasses was the sole carbon source. To address this, two different carbon sources (glucose or glucose + molasses) were tested. When glucose was used, TAPS production reached 2.512 g/L, while glucose + molasses increased TAPS yield to 2.895 g/L, demonstrating that molasses supplementation enhanced TAPS production. Furthermore, with glucose + molasses as the carbon source, the mutant strain achieved a TAPS production rate of 169.5 mg/L/h, 5.5 times higher than before optimization. Finally, high-performance liquid chromatography (HPLC) analysis of the fermentation products revealed the presence of other compounds (reported as minor components produced by *W. ciferrii*). Electrospray ionization mass spectrometry identified one of these compounds as triacetylphytosphingosine (TriAPS), which can also serve as a Cer precursor. In summary, the final yield of Cer precursors, including TAPS and TriAPS, reached 5.114 g/L, 18 times higher than the parent strain (0.284 g/L), 10.5 times higher than the initially reported TAPS yield of the F-60-10 strain (0.485 g/L), and 2.5 times higher than the yield of the CSS.L4O.L2.L1.S2 mutant strain (2 g/L).

Choi et al. obtained a high-yield mutant strain 736 through irradiating the wild-type strain of *W. ciferrii* with γ-rays [62]. Both the mutant strain 736 and the wild-type strain were cultured in shake flasks using four different media (YMgI, YMgIS, YMgIC, and YMgISC) with glycerol (10 g/L) as the carbon source. The results showed that in all four media, both the mutant 736 and the wild-type strain achieved the highest TAPS production in YMgISC medium, with yields of 892 ± 97 mg/L and 442 ± 27 mg/L, respectively. Notably, the TAPS yield of mutant 736 in YMgISC medium was twice that of the wild-type strain. Moreover, the glycerol concentration also has effects on TAPS production. Two initial glycerol concentrations, 35 g/L and 130 g/L, were tested in bioreactor fermentations. When cultured in YMgISC medium with 35 g/L glycerol, mutant 736 produced up to 1.2 g/L of TAPS within 48 h, while the wild-type strain produced 0.8 g/L of TAPS in the same period. In batch fermentation with 130 g/L glycerol, mutant 736 produced 9.1 g/L of TAPS within 120 h, whereas the wild-type strain produced only 1.7 g/L of TAPS. As a result, when cultured in YMgISC medium with a glycerol concentration of 130 g/L in shake flasks, mutant strain 736 achieved the highest TAPS yield of 9.1 g/L, which is 32 times higher than the yield of the parent strain (0.284 g/L) and 18.8 times higher than the initially reported TAPS yield of the F-60-10 strain (0.485 g/L).

#### 3.1.2. Genetic Engineering to Enhance TAPS Production

**Figure 1:**
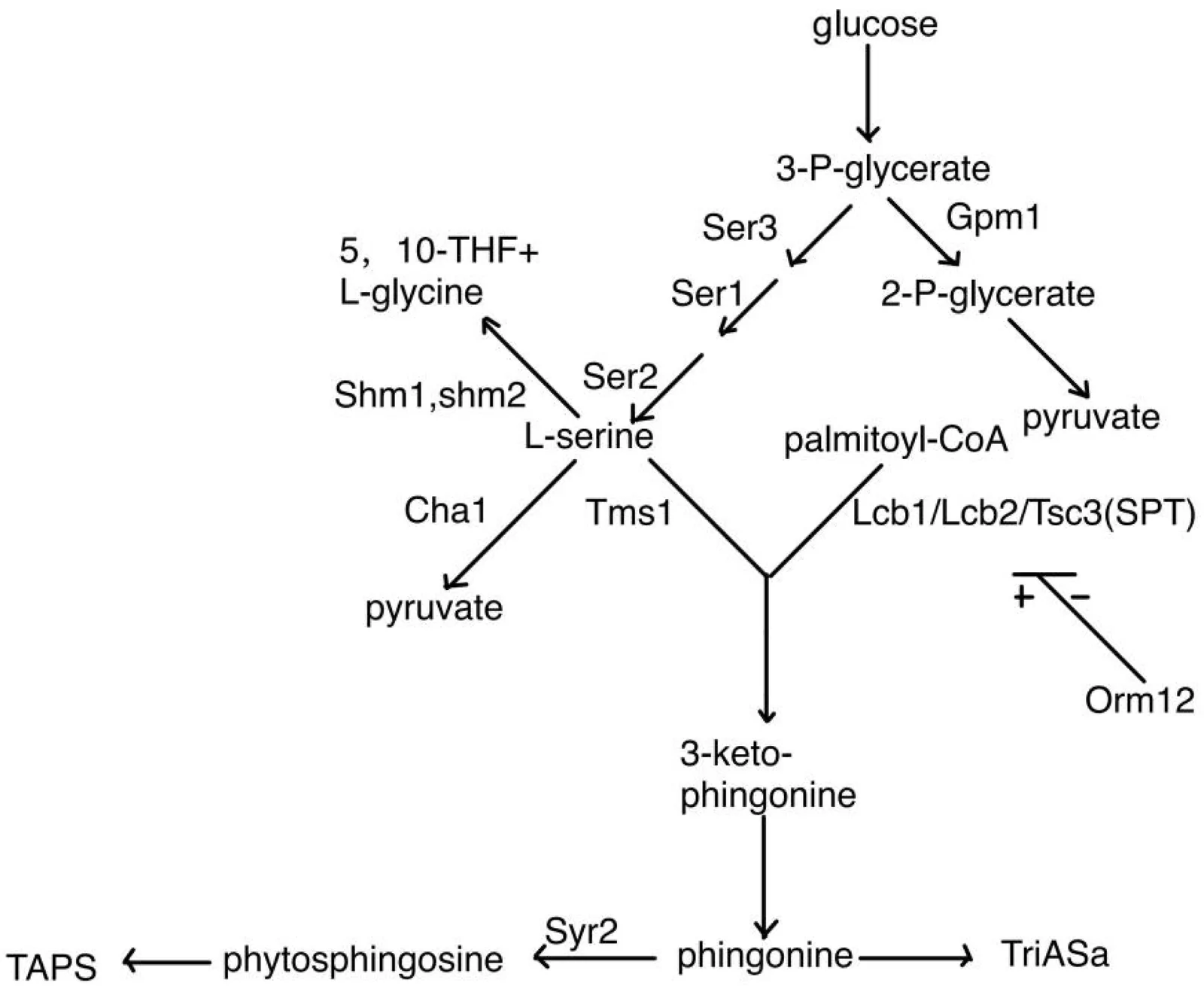
The biosynthetic pathway of Phyto-Sph in *P. ciferrii*.

By modifying genes involved in the synthesis of Phyto-Sph in *P. ciferrii*, an efficient microbial expression system can be constructed to achieve high-yield production of TAPS. Additionally, this approach can improve the growth rate of the strain and enhance its adaptability to environmental conditions. Schorsch et al. genetically engineered a mutant strain of *P. ciferrii* capable of producing large quantities of TAPS. They increased substrate availability by deleting genes involved in the degradation of TAPS precursors, thereby improving sphingolipid biosynthesis flux [63]. The process of Phyto-Sph synthesis in *P. ciferrii* (Figure 1) involves multiple steps and genetic engineering strategies [64]. The first step in the de novo synthesis of TAPS is the condensation of L-serine and palmitoyl-CoA to form 3-ketosphinganine, a reaction catalyzed by serine palmitoyl transferase (SPT). The SPT-catalyzed reaction has been identified as a critical step in Sph synthesis. In this step, Schorsch et al. improved precursor availability by blocking the degradation of L-serine. They deleted two genes—*Shm1* and *Shm2* (encoding L-serine transhydroxymethylase)—resulting in a strain that produced 65 mg/g (dry weight) of TAPS. The second step involves the conversion of 3-ketosphinganine into sphinganine and Phyto-Sph. Here, Schorsch et al. disrupted LCB phosphorylation by deleting the LCB kinase gene *Lcb4*, which increased TAPS production by 78%. Additionally, Phyto-Sph and sphinganine precursors were significantly secreted in their triacetylated forms (TriASa). The overexpression of the two serine palmitoyl transferase subunits, *Lcb1* and *Lcb2*, along with the deletion of *Orm12* (a gene encoding a putative negative regulator of sphingolipid synthesis), resulted in a strain producing 178 mg/g (dry weight) of TAPS. Optimizing the activity of dihydrosphingosine hydroxylase (Syr2) to convert Sph into Phyto-Sph reduced TriASa production and further enhanced TAPS yield. The final recombinant *P. ciferrii* strain achieved TAPS production of up to 199 mg/g (dry weight). The metabolically engineered mutant strain CSS.L4O.L2.L1.S2 produced up to 2.0 g/L of TAPS. Finally, Phyto-Sph undergoes N- and O-acetylation reactions before being secreted outside the yeast cells. The synthesis of Phyto-Sph in *P. ciferrii* involves the regulation of L-serine metabolism, genetic engineering of the sphingolipid synthesis pathway, and the modification and optimization of Phyto-Sph. Through the combined application of these strategies, efficient production of Phyto-Sph was achieved.

In yeast, Phyto-Sph is synthesized via C4 hydroxylation mediated by Syr2. Sph and Phyto-Sph are phosphorylated by Lcb4, then enter the salvage pathway to produce ethanolamine phosphate and aliphatic aldehyde or are converted to Cer through N-acylation [65]. *W. ciferrii* naturally secretes a large amount of acetylated sphingolipid bases, particularly a fully acetylated form of Phyto-Sph known as TAPS. This unique characteristic positions *W. ciferri* as an attractive candidate for the industrial production of TAPS [66]. Additionally, *Yarrowia lipolytica* (*Y. lipolytica*) is also one of the promising hosts for sphingolipid production, which could be a preferred safe biotechnological microorganism for producing lipid-derived chemicals [67]. Han et al. successfully constructed a *Y. lipolytica* CE3 strain that produces TAPS by co-expressing the acetyltransferases SLI1 and ATF2 derived from *W. ciferri*. Relevant culture conditions were further optimized in shaking culture to enhance TAPS yield [68]. The maximum yield of TAPS was 106.15 mg/L at an optimal cultivation temperature of 28 °C. The deletion of the LCB4 gene in the CE3 strain increased the TAPS yield to 142.1 ± 10.7 mg/L. NaOH and NH_4_OH were used as pH regulators and compared, both utilizing glycerol as a carbon source. When NH_4_OH was used to control pH, NH_4_^+^ remained above the initial value, with a concentration of 216.7 mmol/L throughout the fermentation. In contrast, when pH was maintained with NaOH, the concentration of NH_4_^+^ dropped to undetectable level (<0.278 mmol/L), resulting in a TAPS yield of 0.25 ± 0.011 g/L after 32 h of fermentation, which was higher than that obtained with NH_4_OH (0.16 ± 0.005 g/L). The evaluation of using vegetable oils (olive oil and palm oil) as carbon sources was conducted, with both cultures set at an initial temperature of 28 °C. However, palm oil solidified at this temperature, and the corresponding culture was prone to denaturation at 35 °C. After 56 h of cultivation, the TAPS yield in the olive oil-containing culture was 0.65 ± 0.024 g/L, which was higher than that in the palm oil-containing culture (0.044 g/L). In summary, through the modification of relevant genes and optimization of conditions, a TAPS yield of 0.65 g/L was achieved, which is 2.3 times that of the parent strain (0.284 g/L) and 1.3 times that of the previously reported F-60-10 strain (0.485 g/L).

#### 3.1.3. Screening of Haploid Strains for High TAPS Production

The principle of haploid screening involves treating cells with appropriate chemicals before cell division to reduce the chromosome number by half, resulting in haploids [69]. Research by Wickerham and his colleagues found that the production of TAPS may be related to the sex of *W. ciferrii* strains. They also discovered significant differences in TAPS production levels among different mating types, suggesting that genetic factors influencing TAPS yield are not solely determined by sex [61,62]. The high-yielding strain NRRL Y-1031, and F-60-10, is one of the mating-type isolates of NRRL Y-1031. Although Y-1031 shares the same sex as F-60-10, it produces significantly less TAPS than F-60-10. Based on these findings, Park inferred that gene recombination during the meiosis of diploid *W. ciferrii* strains leads to the formation of haploid spores, resulting in a new haploid mating type strain (DSCC7-25) [70]. To isolate high-yielding strains from the spore pool, Park added a selective chelator (ethylene glycol tetraacetic acid) to calcium ions in the culture medium. Calcium ions have been shown to influence sphingolipid biosynthesis by regulating the activity of key enzymes involved in the pathway. The incorporation of this chelator depletes calcium ions, thereby inhibiting yeast cell growth. It is speculated that new haploid strains capable of growing in these selective environments may be high-yielding strains. To further enhance TAPS yield, researchers optimized fermentation conditions, determining the optimal parameters to be a temperature of 25 °C, an agitation speed of 210 r/min, and a pH of 7.5. Using a fed-batch fermentation mode, glycerol was gradually added to the fermentation broth, ultimately achieving a TAPS yield of 14 g/L. This modification significantly increased TAPS production, approximately 29 times that of the previously reported yield for the F-60-10 strain (0.485 g/L).

The above description outlines three methods for screening high-yield TAPS-producing strains: mutagenesis breeding, genetic engineering, and haploid strain screening. Although genetic engineering offers strong targeting, high modification precision, and the ability to precisely optimize metabolic pathways, it is associated with issues such as high technical barriers and high operational costs [71]. Haploid breeding, while enabling rapid homozygosity of genetic traits and improving screening accuracy, is limited to a narrow range of applicable strains and is often used as an auxiliary breeding method, making it difficult to independently achieve large-scale high-yield improvements [72]. In contrast, mutagenesis breeding is simple, offers a broad mutation spectrum, and has a relatively short screening period, making it the preferred method for efficient short-term strain development [73]. Nevertheless, strain-endogenous metabolic pathways exclusively yield Phyto-Sph and cannot synthesize the three long-chain-base skeletons required by human epidermis: sphingosine, dihydrosphingosine, and 6-hydroxysphingosine. Accordingly, the complete profile of 12 types of native stratum-corneum Cer cannot be reconstructed, resulting in structural defects in compatibility with endogenous skin lipids. Post-TAPS deacetylation, the subsequent ex vivo amidation process proceeds devoid of acyl-chain chemoselectivity, resulting in C16 and C18 medium-short-chain Cer. In accordance with the preceding elaborated structure–activity relationships, excess medium-short-chain Cer would disrupt ordered intercorneocyte lamellar assemblies, instigating mitochondrial functional aberration and intracellular ROS overloading, which synergistically amplify cutaneous oxidative burden and pro-inflammatory signaling cascades. As a corollary, fermented blends manifest conspicuous inter-batch variance in barrier rehabilitation and radical-scavenging bioactactivities, failing to conform to quantitative quality control thresholds stipulated for bioactive cosmetic feedstocks. From the perspective of process industrialization, the reported maximal TAPS titre of 22.10 g/L is solely replicable under 5 L lab-scale bioreactor cultivation regimens. Scaling cultivation systems to hectoliter pilot bioreactors impedes the coordinated orchestration of microbial vegetative growth and sphingolipid anabolism, engendering batch-to-batch concentration deviations surpassing 35% and undermining manufacturing reproducibility. The high-glycerol fed-batch fermentation paradigm discharges high-chemical-oxygen-demand organic wastewater; ancillary three-waste remediation infrastructure imposes substantial increments in environmental governance and operational overheads, severely impeding industrial translatability of this biomanufacturing pipeline.

### 3.2. Natural Extraction of Ceramides

With the continuous optimization of biotechnology, the yield of TAPS produced by *W. ciferrii* has been steadily increasing. However, the yield remains low, and microbial fermentation can only obtain the precursor substance of Phyto-Sph—TAPS. Therefore, to directly acquire various Cer, researchers have applied extraction techniques to derive Cer from various natural biological materials.

#### 3.2.1. Extraction of Ceramides from Animals

**Figure 2:**
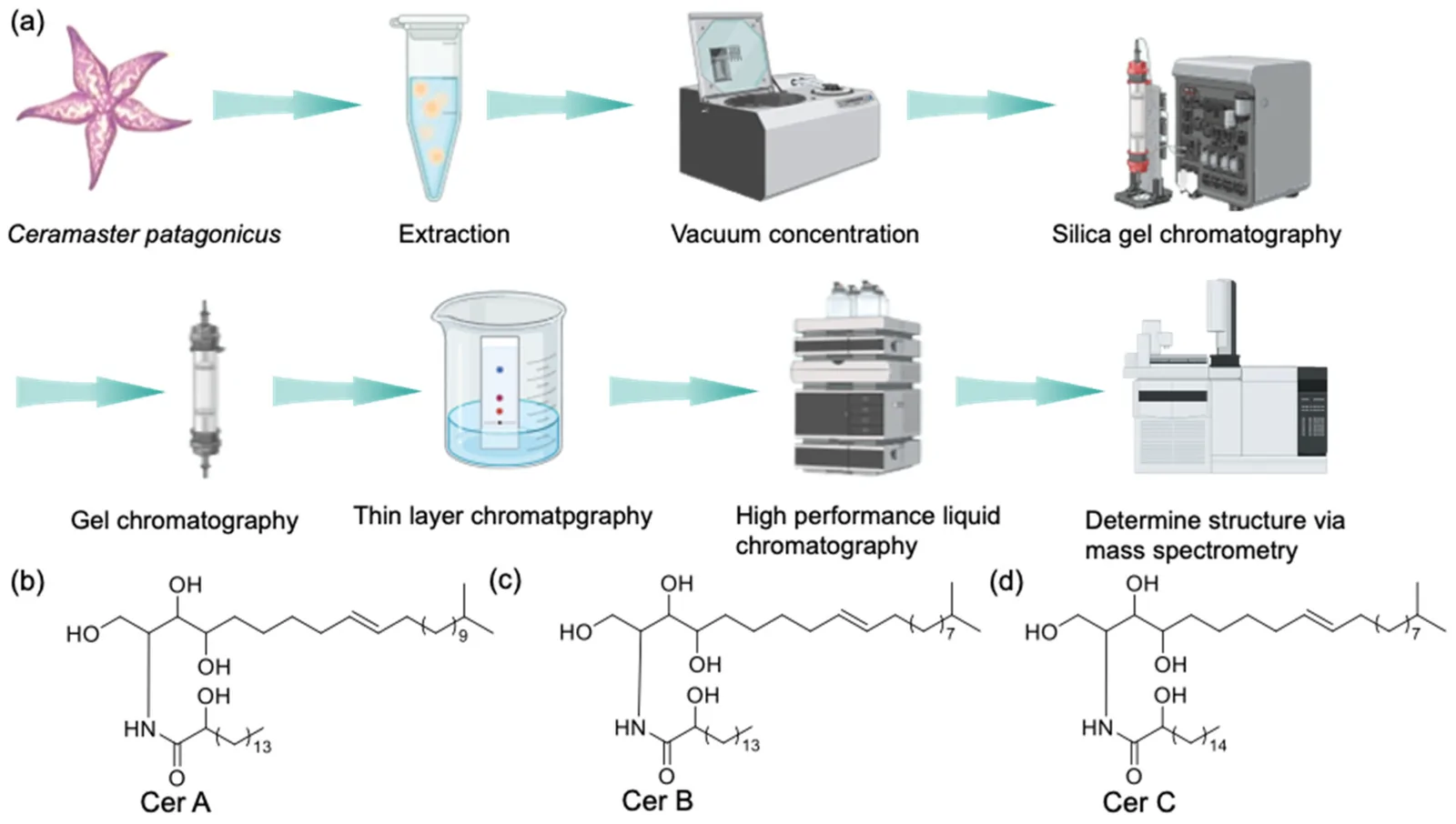
(**a**) Experimental procedure for extracting three types of Cer from *Ceramaster patagonicus*. (**b**–**d**) The chemical structures of Cer A-C. Created in BioRender. Yan, Y. (2026) https://BioRender.com/s9d4ryo.

Starfish are found in oceans around the world, and their ecological environments and life history traits make them a rich source of various low molecular weight compounds. Studies have found multiple sphingolipids in starfish [74]. Malyarenko and colleagues obtained three Cer varieties from the Far Eastern deep-sea starfish *Ceramaster patagonicus* [75]. As shown in Figure 2, fresh slices of *C. patagonicus* (3 kg) were extracted using CHCl_3_:MEOH = 2:1, followed by further extraction with CHCl_3_:MEOH = 1:1 and EtOH. The combined extracts were vacuum-concentrated to a residue of 159.5 g. This residue was then separated in a mixed solvent of AcOEt:BuOH = 2:1 and H_2_O, with the organic layer vacuum-concentrated to obtain a less polar fraction (51.5 g), which was eluted with cold acetone. The acetone-soluble fractions were subjected to chromatographic separation on a column using CHCl_3_, CHCl_3_:MEOH = 97:3, and CHCl_3_:MEOH = 9:1, yielding four fractions: 1, 2, 3, and 4. Subsequently, fractions 1–4 underwent further chromatographic analysis on a gel column using a step gradient of n-hexane, namely AcOEt:BuOH (6:3:0.1 → 6:3:0.7, *v*/*v*/*v*), producing six sub-fractions: 21, 31, 32, 41, 42, and 43. Thin-layer chromatography was conducted on the elution system using CHCl_3_:MEOH:H_2_O = 8:1:0.1 gradient. Using MeOH as the eluent, high-performance liquid chromatography (HPLC) was performed on sub-fraction 31, resulting in the isolation of Cer A (12 mg) along with 17 sub-fractions (31-3 to 31-15 and 17-31 to 20-31). HPLC was also carried out on sub-fractions 31-14 and 31-18 using MeOH, yielding Cer B (2 mg) and unseparated Cer A and C (1.5 mg). Finally, mass spectrometry was employed to determine the structures of the three Cer, which were composed of unsaturated sphingosine and α-hydroxy fatty acids with 16 or 17 carbons (with a total extraction rate of 0.005%).

**Figure 3:**
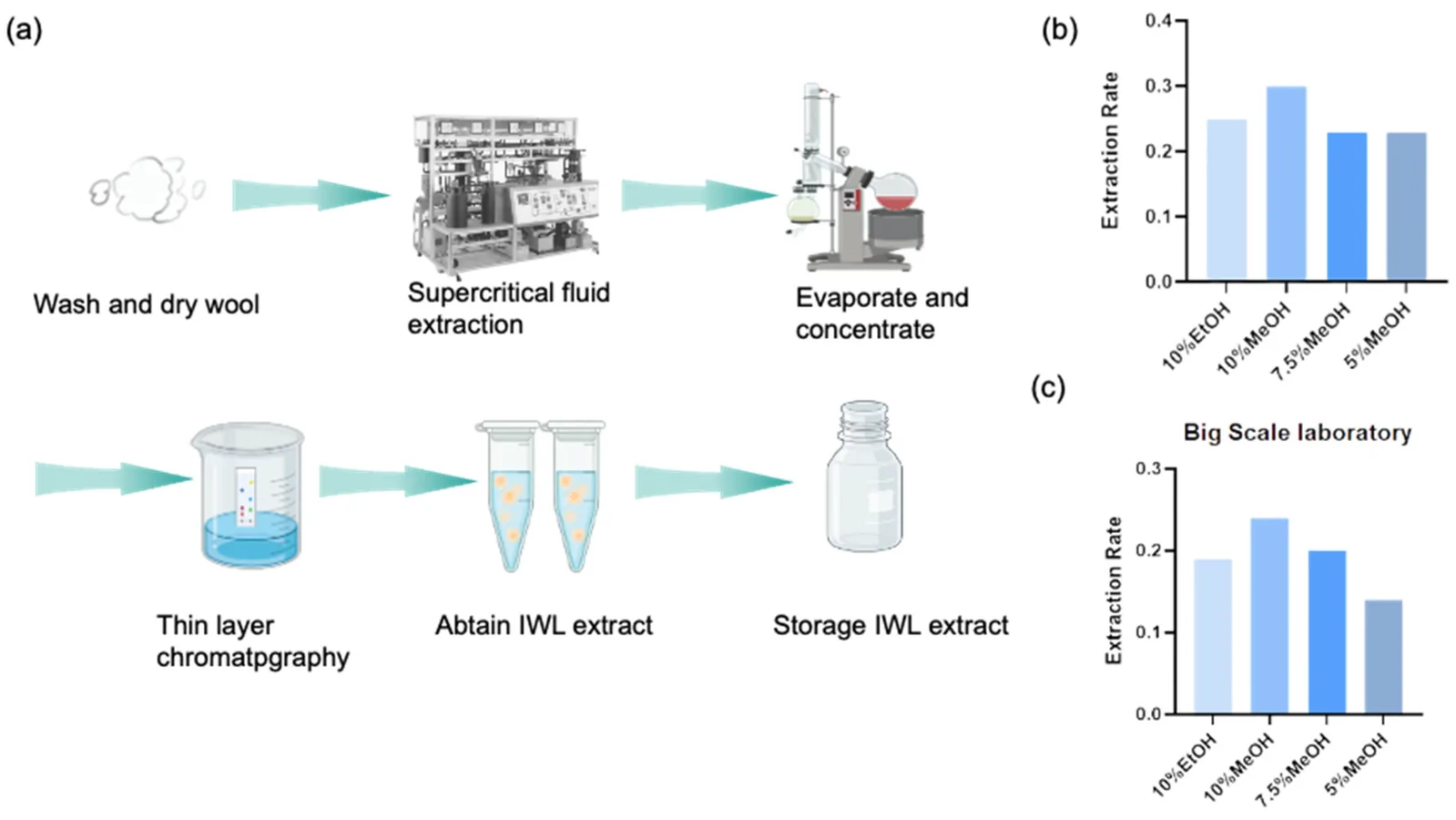
(**a**) Experimental procedure for extracting Cer from wool. (**b**) Small-scale laboratory extraction rates. (**c**) Large-scale laboratory extraction rates. Created in BioRender. Yan, Y. (2026) https://BioRender.com/8uzd4fe.

Supercritical Fluid Extraction (SFE) is a novel extraction and separation technique that utilizes supercritical fluids as solvents to extract specific components from liquids or solids for separation purposes [76]. Wool is a natural protein fiber primarily composed of keratin, containing external lipid content (lanolin) and a small amount of internal lipid content (1.5%). These internal wool lipids (IWL) are rich in cholesterol, free fatty acids, cholesterol sulfate, and Cer, with a composition similar to that found in other keratinous tissues such as human hair or skin stratum corneum. When applied to the skin, IWL can improve skin condition [77,78]. Therefore, IWL extracts with a liposomal structure are suitable for incorporation into drug or cosmetic formulations for skin treatment and care. To enhance the extraction yield of Cer, Ramirez et al. employed SFE instead of traditional extraction methods to obtain IWL extracts rich in Cer from wool (Figure 3) [79]. First, the wool samples were industrially cleaned using sodium carbonate and non-ionic polyvinyl fluoride surfactants, followed by rinsing with water and mechanically agitating to remove any residual plant materials, and then the wool was dried. Subsequent extraction experiments were conducted at both small and large laboratory scales. For the small-scale experiments (SFE equipped with a 50 mL extraction vessel), each control experiment filled the extraction vessel with 10 g of wool. Ethanol and methanol (at concentrations of 10.0%, 7.5%, and 5.0%) were used as modifiers and delivered to the system. The experiments were conducted under varying pressures (160, 200, and 250 atm), temperatures (40, 50, and 60 °C), and CO_2_ volumes (200, 250, 300, and 350 mL). The extracts were collected into capped vials using a fixed flow restrictor system, and the vials were hermetically sealed to expel the depressurized CO_2_. The extracts were concentrated to dryness under a gentle nitrogen flow and quantified by weight analysis. For the large-scale experiments (SFE equipped with a 375 mL extraction vessel), each control experiment filled the vessel with 50 g of wool. Ethanol and methanol (at concentrations of 10.0%, 7.5%, and 5.0%) were again used as modifiers. The experiments were performed at different pressures (160 and 200 atm) and temperatures (40 and 60 °C), with CO_2_ volumes of 200, 250, and 350 mL used for each extraction, and the extracts were collected until environmental conditions were reached. The extracts were concentrated to dryness using a vacuum concentrator and quantified by weight analysis. The IWL extracts obtained from both laboratory scales were stored in chloroform/methanol (2:1, *v*/*v*) solvent at 20 °C. This technique enables rapid separation and precise quantification of different lipid classes without the need for sample pre-treatment, allowing for the study of the composition of various lipid extracts. Before conducting a full scan of the lipids, the extracts were eluted twice with chloroform/methanol/water (57:12:0.6, *v*/*v*/*v*) to separate Cer. The extraction results for total substances and Cer are shown in Figure 3 and Table 2, indicating that the highest ceramide extraction rate was achieved at a temperature of 60 °C, pressure of 160 atm, and CO_2_ volume of 250 mL, with 10% methanol as the modifier. The extraction rates for the small and large laboratory scales were 0.30% and 0.24%, respectively.

The extraction rates of Cer from the two animals mentioned above differ significantly. This discrepancy may be attributed to the varying percentages of Cer content in the two animals. Additionally, another reason could be the optimization of techniques used during the extraction of Cer from wool, which helps to minimize losses in the extraction process.

#### 3.2.2. Extraction from Plants

**Figure 4:**
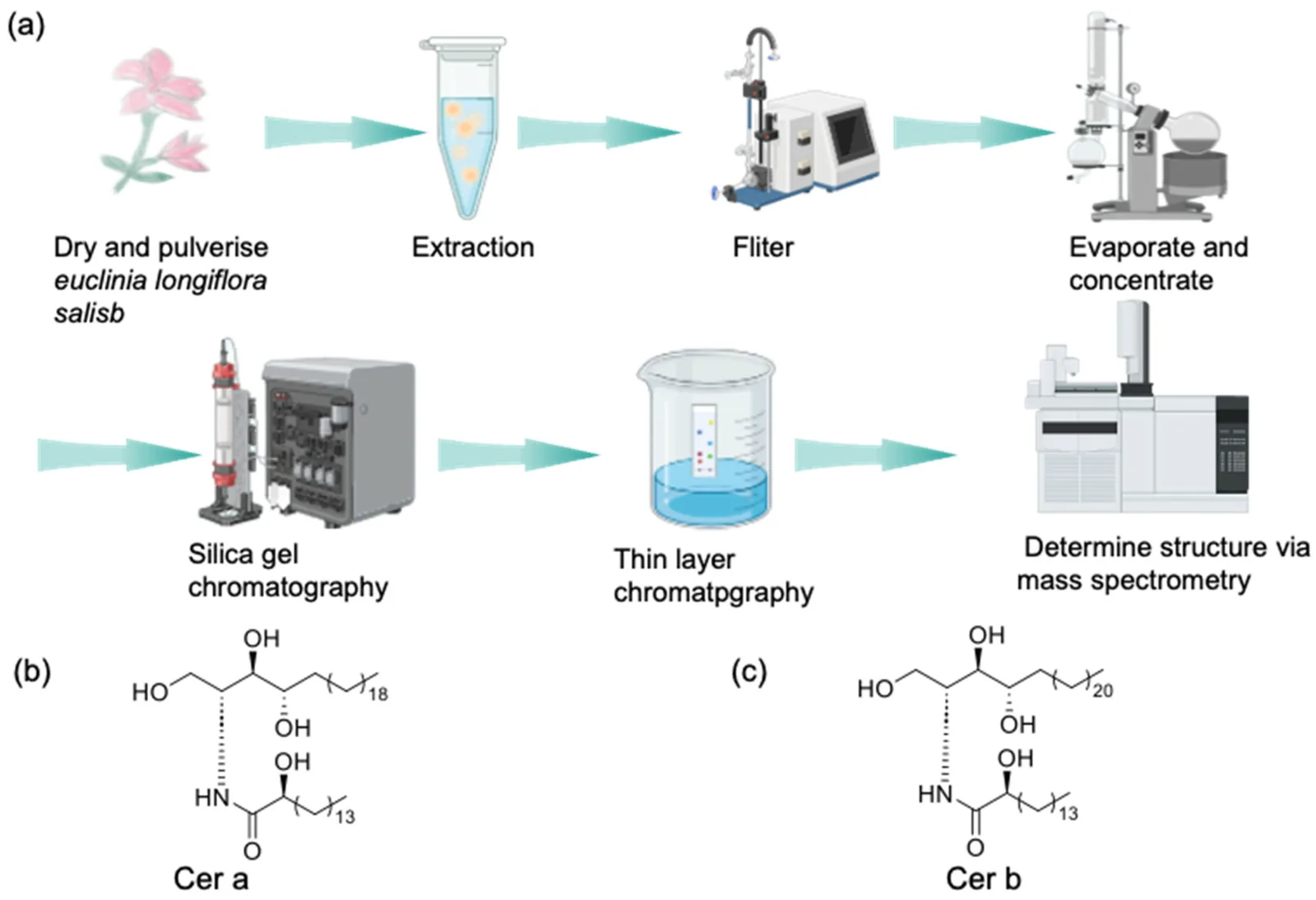
(**a**) Experimental procedure for extracting Cer from *Euclinia longiflora Salisb*. (**b**) Chemical structure of Cer a. (**c**) Chemical structure of Cer b. Created in BioRender. Yan, Y. (2026) https://BioRender.com/fikzpxl.

Due to the disease risks associated with Cer from animal sources, researchers have begun to consider extracting Cer from plants. *Euclinia longiflora Salisb.*, a member of the *Parinari* family, is primarily distributed in tropical and subtropical regions of the world. Different parts of this plant have been used in traditional medicine to treat diseases such as malaria and skin conditions [80,81]. Munvera et al. extracted two Phyto-Sph-type Cer from the leaves of *Euclinia longiflora Salisb*, which have therapeutic effects against skin and subcutaneous parasitic infections [82]. As shown in Figure 4, the leaves were dried in a cool place at room temperature, then ground into powder. The powder was soaked in methanol three times at room temperature for extraction. The resulting suspension was filtered, followed by concentrating under vacuum using a rotary evaporator, yielding a brown residue. The methanol extract was fractionated on silica gel using a hexane/ethyl acetate gradient, resulting in three main fractions: S1 (4:1 hexane/ethyl acetate), S2 (1:1 hexane/ethyl acetate), and S3 (pure ethyl acetate). Fraction S1 was subjected to chromatographic separation on a silica gel column, eluted with a gradually increasing polarity mixture of hexane/ethyl acetate, and 142 fractions were collected. These fractions were further separated using thin-layer chromatography (TLC), and based on the TLC results, five main sub-fractions were obtained: T1 (1–30), T2 (31–60), T3 (61–86), T4 (87–115), and T5 (116–142). Among these, sub-fraction T4 was purified to yield two Phyto-Sph-type Cer, and their structures are shown in Figure 4b,c. Ultimately, 2 mg of Cer a and 5 mg of Cer b were extracted from 0.6 kg of *Euclinia longiflora Salisb* leaves, resulting in an extraction rate of 0.0012%.

**Figure 5:**
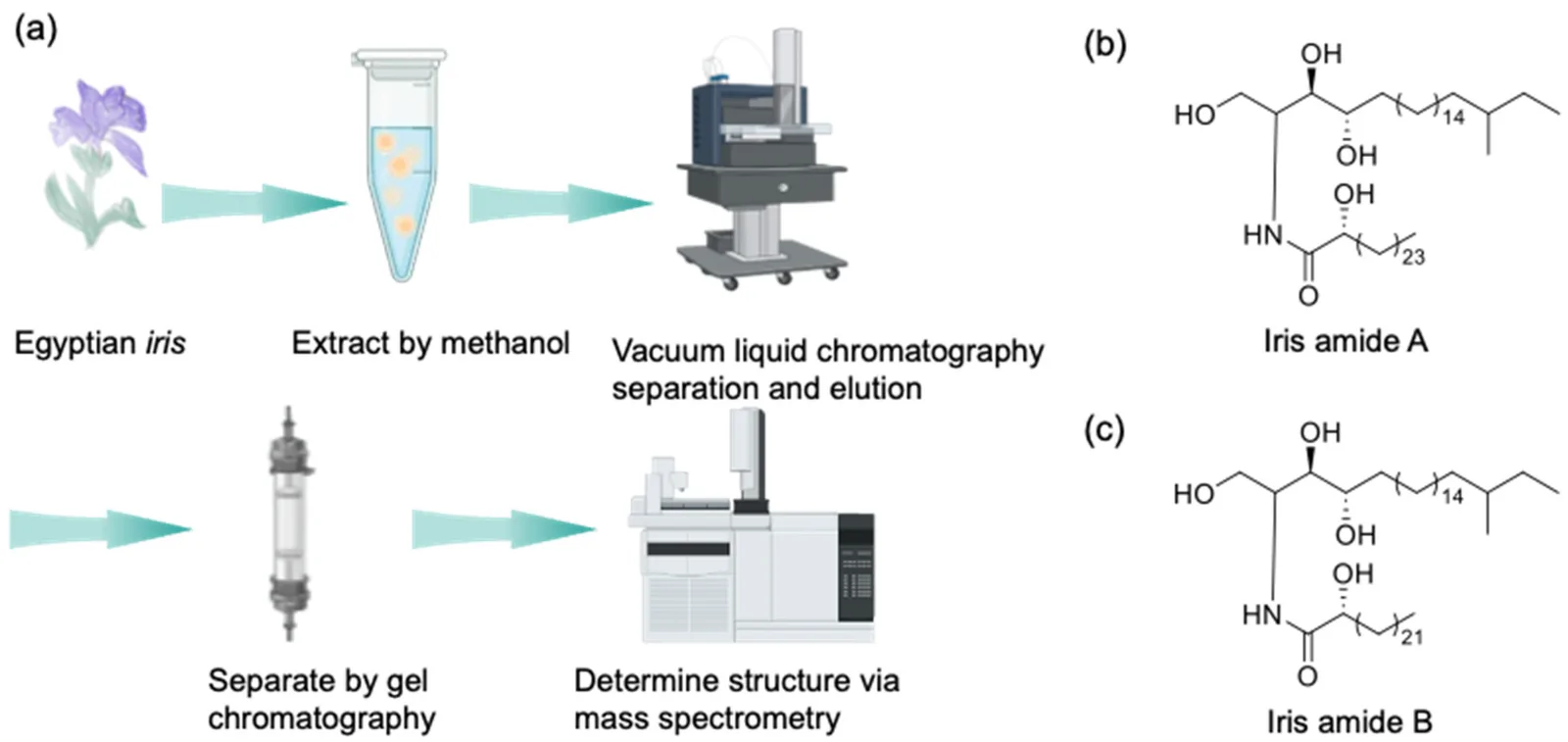
(**a**) Experimental procedure for extracting two new Cer with ultra-long-chain from *Egyptian Iris* rhizome powder. (**b**) Iris amide A. (**c**) Iris amide B. Created in BioRender. Yan, Y. (2026) https://BioRender.com/250gvqn.

Mohamed et al. extracted two new ultra-long-chain Cer from the rhizome powder of the *Egyptian Iris* (Figure 5) [83]. At room temperature, they used 70% methanol to extract 320 g of dried rhizome powder. The methanol extract was then subjected to vacuum liquid chromatography (VLC) using hexane, CHCl_3_, EtOAc, and MeOH, resulting in four fractions (hexane IG-1, CHCl_3_ IG-2, EtOAc IG-3, and MeOH IG-4). Next, fraction IG-1 was subjected to gradient elution using hexane/EtOAc as the solvent, yielding four sub-fractions: IG-1-A (100% hexane), IG-1-B (hexane: EtOAc, 75:25), IG-1-C (hexane: EtOAc, 50:50), and IG-1-D (100% EtOAc). Sub-fraction IG-1-D was further purified multiple times on a silica gel column using a hexane/EtOAc gradient, resulting in two new Cer, named Iris amide A and Iris amide B (their chemical structures are shown in Figure 5b,c). The total mass was 41 mg, with an extraction rate of 0.128%.

**Figure 6:**
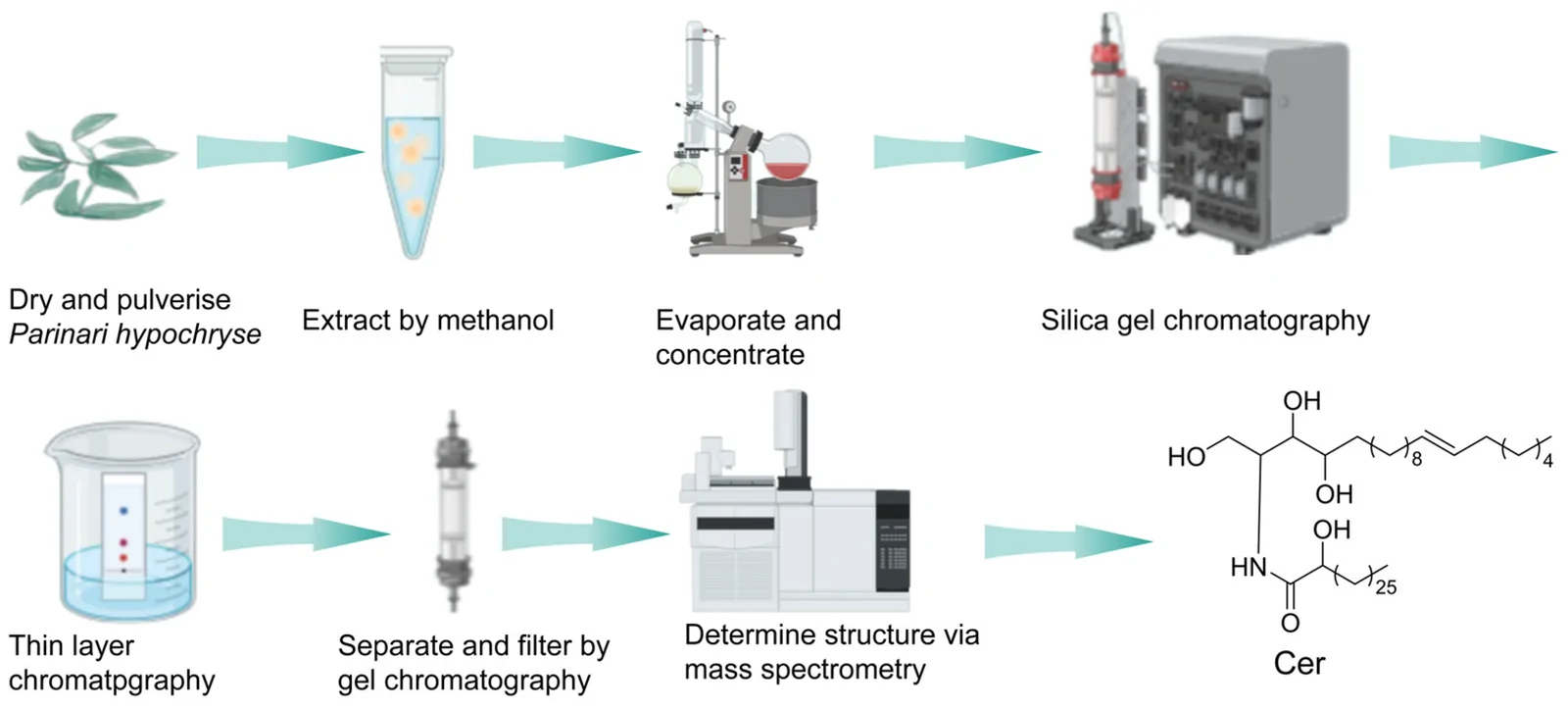
Experimental procedure for extracting Cer from *Hypochrysea* and the chemical structure of the obtained Cer. Created in BioRender. Yan, Y. (2026) https://BioRender.com/tahkbo7.

Members of the *Parinari* genus are distributed globally and are used to treat various conditions such as diabetes, respiratory difficulties, malaria, toothaches, and venous diseases [84,85,86]. Adjapmoh et al. extracted Cer from *Hypochrysea*, which belongs to the *Parinari* plant (Figure 6) [87]. The leaves of *Hypochrysea* were sun-dried and then ground into a powder (1.81 kg) followed by extracting in methanol. The extract was evaporated, yielding a crude residue of 183.3 g. From this residue, 150 g were fractionated using silica gel VLC with solvents in the following order: hexane, CH_2_Cl_2_, ethyl acetate, CH_2_Cl_2_:MeOH (9:1), and MeOH; this resulted in fractions A (0.5 g), B (9.4 g), C (12.9 g), D (35.0 g), and E (82.0 g). TLC was used for analysis, and fractions C and D were combined (47.9 g). These were then subjected to silica gel column chromatography using a mixed solvent of CH_2_Cl_2_ and CH_2_Cl_2_:MeOH, with MeOH concentration gradually increased from 2.5% to 100%. This process generated eight fractions (F1–F8). Sub-fractions F3 and F5 were obtained from CH_2_Cl_2_:MeOH (95:5) and CH_2_Cl_2_:MeOH (92.5:7.5), respectively, and were subjected to gel filtration on hydroxypropyl cellulose gel, eluting with MeOH. A pure Cer was obtained from CH_2_Cl_2_:MeOH (95:5) (45 mg), and its structure was determined by mass spectrometry, as shown in Figure 6. The extraction rate of Cer was approximately 0.0025%.

**Figure 7:**
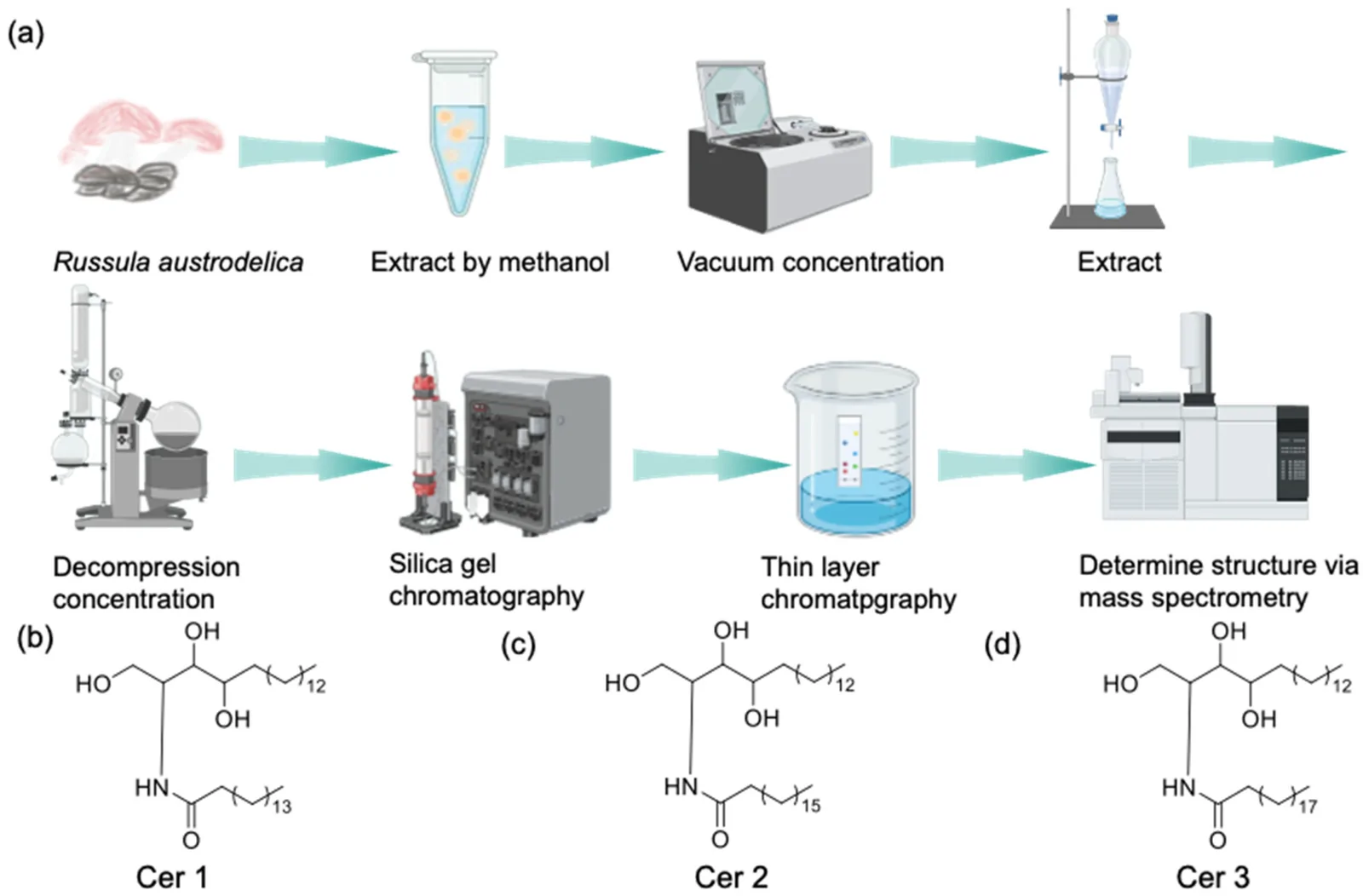
(**a**) Experimental procedure for extracting Cer from *Russula austrodelica*. (**b**–**d**) Structural formulas of the obtained Cer. Created in BioRender. Yan, Y. (2026) https://BioRender.com/yt8glgs.

Reports indicate that the genus *Russula* contains Cer [88,89]. Alarcon et al. extracted three types of Cer from *Russula austrodelica* [90]. As illustrated in Figure 7, methanol was first used to extract the fresh *Russula austrodelic* (683 g) five times at room temperature. The methanol extract was then filtered and concentrated under vacuum at 40 °C and 180 mb, yielding a crude residue of 85 g. The methanol extract was subjected to solvent partitioning in a MeOH/H_2_O mixed solvent, transferred to a separatory funnel, and extracted with hexane. The hexane phase was combined and concentrated under reduced pressure. The same process was repeated using ethyl acetate to obtain the ethyl acetate fraction, which was then concentrated to yield a residue. This residue was subjected to silica gel chromatography, eluted with a mixture of CH_2_Cl_2_:MeOH (10:1), and finally purified using TLC, resulting in Cer (3 g). Mass spectrometry determined that there are three types of Cer (Cer1-3), and their structural formulas are shown in Figure 7b-d. The extraction rate of Cer was approximately 0.44%.

**Figure 8:**
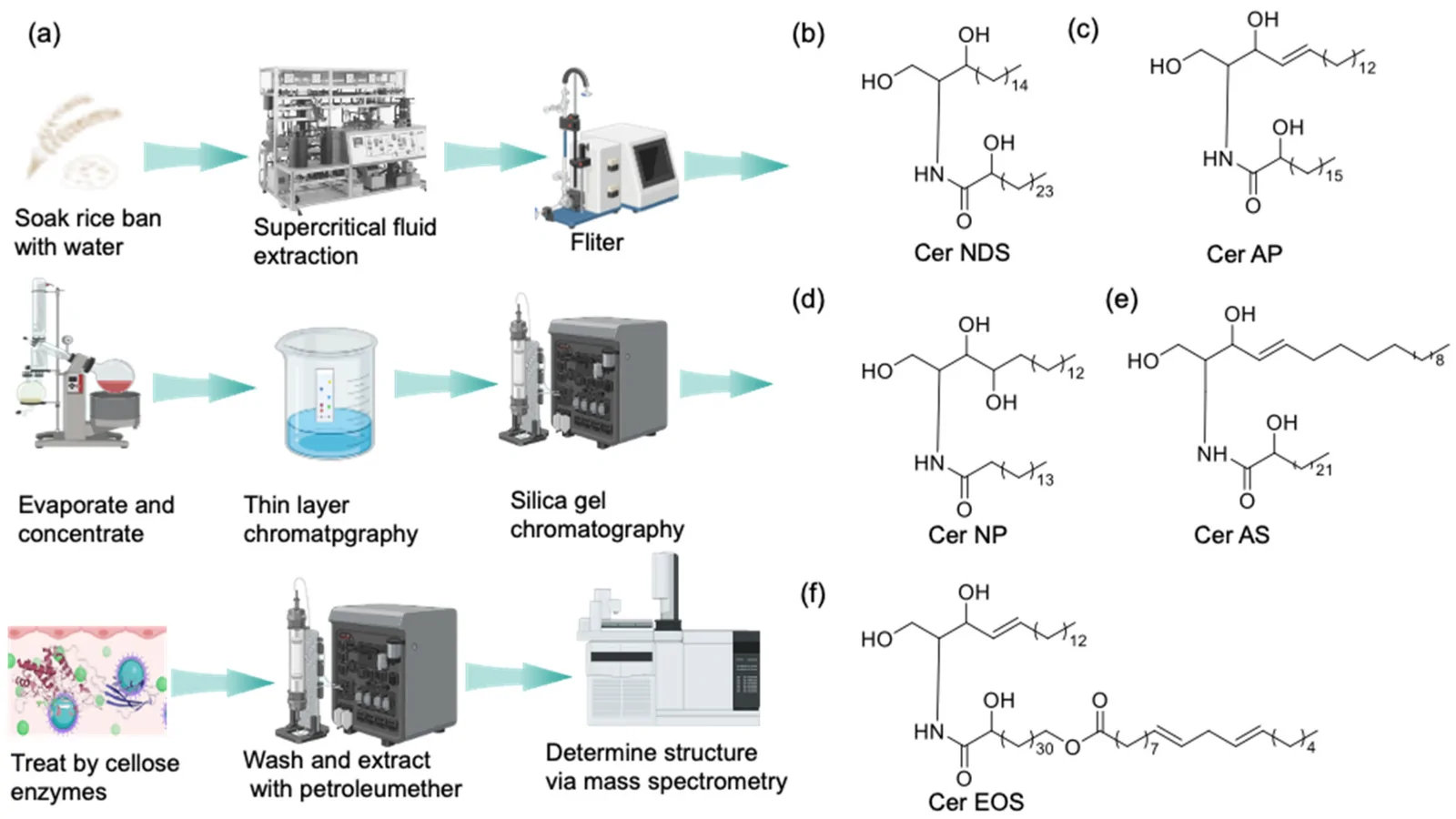
(**a**) Experimental procedure for extracting Cer from rice bran. (**b**–**f**) Five types of the obtained Cer. Created in BioRender. Yan, Y. (2026) https://BioRender.com/jd6zg0h.

The extraction of Cer from the aforementioned four plants relied solely on different solvents, leading to partial loss of Cer during the extraction process. Ultrasonic-assisted extraction technology utilizes the physical effects of ultrasound to accelerate solvent penetration into the sample, allowing for the rapid and efficient extraction of target compounds from the sample [91]. Response Surface Methodology (RSM) is a modeling technique that combines mathematical and statistical methods to optimize experimental conditions [92]. Sea rice, a novel salt-tolerant rice variety, contains abundant minerals, proteins, and lipids, particularly its byproduct, rice bran, which can serve as a primary source of Cer, replacing other commercial sources like rapeseed [93]. Wang et al. extracted Cer from the rice bran of sea rice, enhancing the extraction rate by combining ultrasonic-assisted extraction with RSM [94]. The experimental procedure is illustrated in Figure 8. Using 95% ethanol, the rice bran was soaked for a certain period. The soaked rice bran was then processed with an ultrasonic extractor. The extract was allowed to stand for 5 min before being heated and filtered under reduced pressure. The resulting extract was concentrated using a rotary evaporator. TLC was employed to identify the rice bran extract, followed by purification and separation using silica gel chromatography. Petroleum ether was added to a separatory funnel, and the solute was treated with 1% cellulase at 42 °C and pH 4.5 for extraction, separating the concentrate from the ethanol. After enzymatic hydrolysis, the extract in the beaker was allowed to stand for 5 min before petroleum ether was added for extraction and elution. Finally, the extract was vacuum-dried until no residual petroleum ether or ethanol remained, yielding the Cer extract. Mass spectrometry was then performed to determine the types and quantities of Cer present. Results identified 46 different types of Cer in sea rice bran, with Cer AP, Cer AS, Cer EOS, Cer NAS, and Cer NS (structural formulas shown in Figure 8b–f) accounting for 23.66%, 17.54%, 14.91%, 11.96%, and 7.32%, respectively. Before conducting RSM, single-factor experiments were used to determine the selection range for RSM. The effects of four single-factor variations on Cer yield were evaluated: solid-to-liquid ratio (1:3, 1:4, 1:5, 1:6, and 1:7), extraction solvents (95% ethanol, petroleum ether, ethyl acetate, chloroform), extraction time (25, 35, 45, 55, and 65 min), and extraction temperature (40, 50, 60, 70, and 80 °C). The optimal experimental conditions were found to be a solid-to-liquid ratio of 1:5, extraction solvent of 95% ethanol, extraction time of 46 min, and extraction temperature of 46 °C, resulting in a Cer extraction rate of 12.54%. The experimental results demonstrated that the ultrasonic-assisted extraction process for Cer from sea rice bran, optimized using RSM, yielded a higher Cer output (12.54%) compared to that obtained by traditional SFE (8.36 ± 0.03%).

The extraction rates of Cer from the aforementioned plants vary significantly, likely due to the differing percentages of Cer present in each plant. The extraction rate of Cer from rice bran is much higher than that from other plants, attributed to the optimization of technology and extraction conditions, which minimized losses during the extraction process. Nevertheless, the pathways for extracting Cer from plants still face the problems of low yield and limited plant sources. Since Cer is insoluble in water, organic solvents must be selected as extraction solvents. In the extraction processes mentioned above, methanol is the most commonly used solvent. Additionally, techniques such as ultrasound-assisted extraction and SFE can also be employed. For the optimization of relevant process parameters, RSM can be used to efficiently identify the optimal operating conditions, thereby reducing experimental costs and time. Endogenous sphingolipids are separated from biological matrices including plant, fungi and animal through solvent maceration, ultrasound-intensified mass transfer and supercritical fluid elution methodologies. Nevertheless, all native extraction pipelines suffer from the pivotal limitation of compositional heterogeneity within crude lipid fractions. Crude eluates concurrently accommodate heterogeneous Cer spanning short, medium and ultra-long acyl carbon chains, and their molar proportions fluctuate dramatically as a function of raw material provenance, phenological cycles and harvesting seasons. Apart from the low extraction efficiency, such prominent inter-batch variability in biological efficacy fails to comply with standardized quantitative quality control criteria for efficacy-targeted dermatological feedstocks. From the perspectives of raw material supply and process sustainability, most medicinal woody plants and wild fungi feature protracted growth cycles, which preclude year-round uninterrupted feedstock provision. Although rice bran, an agro-industrial by-product, boasts abundant yields, its crude lipid extracts encompass intricate impurity profiles. Multi-stage chromatographic polishing is indispensable for the elimination of interfering contaminants, which substantially escalates downstream purification costs. Conventional extraction procedures extensively adopt halogenated hydrocarbons and short-chain alcohols with intrinsic toxic potential, introducing risks of solvent residues that violate the safety thresholds for pharmaceutical and cosmetic raw materials. Despite supercritical CO_2_ extraction eliminating residual solvent hazards, the massive capital investment required for high-pressure reaction vessels renders this technique economically infeasible for small- and medium-scale manufacturers. Animal-derived sphingolipid substrates incur irreparable biosafety hazards including zoonotic pathogens and marine biotoxin contamination, rendering such extracts disqualified against regulatory access standards for medical dressings and premium dermocosmetics. Collectively, Cer acquired via natural extraction are only applicable to low-cost cleansing and moisturizing commodities labeled with “natural origin” without quantitative claims of barrier restitution or anti-photoaging efficacy. They are incapable of satisfying stringent feedstock specifications on sphingolipid purity and bioactivity homogeneity stipulated by high-performance cutaneous remedial preparations.

### 3.3. Chemical Synthesis of Ceramides and Related Compounds

Due to the limitation of obtaining only the precursor of Phyto-Sph (TAPS) through microbial fermentation and the low extraction yield from natural sources, researchers have begun to actively explore chemical synthesis. This approach takes advantage of the benefits of chemical reactions—such as ease of operation, simplicity, suitability for large-scale production, high yield, and low raw material costs—greatly increasing the yield of Cer. Additionally, chemical synthesis allows for the production of various ceramide precursors and their derivatives.

#### 3.3.1. Acylation Coupling Reaction of Fatty Acids and Long-Chain Bases

**Figure 9:**
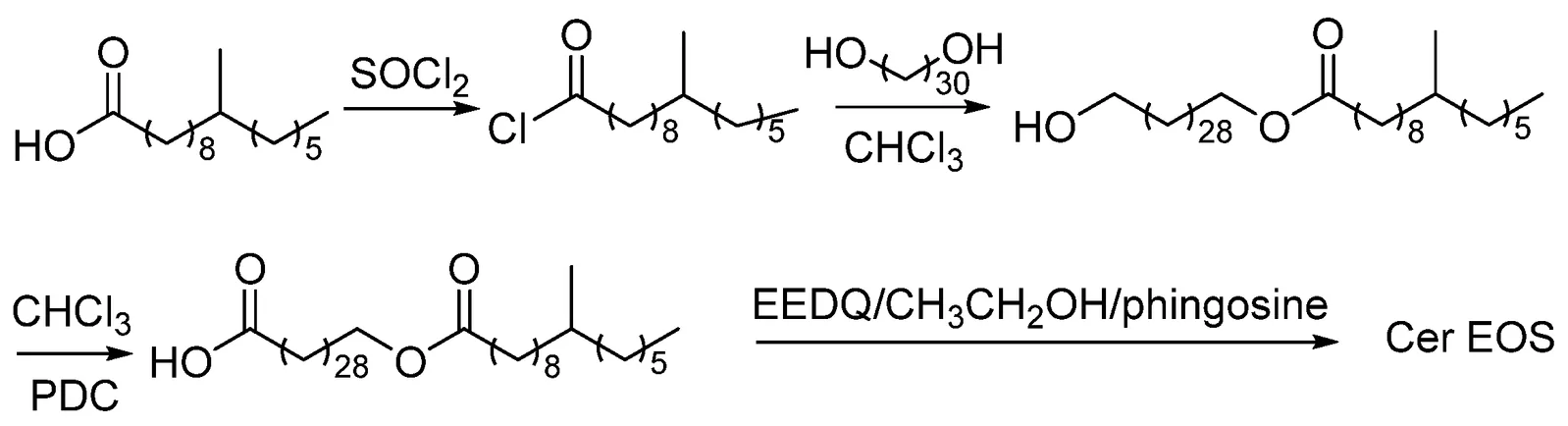
Synthesis of Cer EOS [95].

Engelbrecht et al. synthesized a novel C10-methyl-branched palmitic acid acylated Cer EOS [95], as shown in Figure 9. The reaction began with the acylation of 10-methylhexadecanoic acid with thionyl chloride to produce 10-methylhexadecanoyl chloride, followed by a reaction with 1,3-diol to generate a monoester. The alcohol hydroxyl group on the monoester was then oxidized to the corresponding acid. Finally, in the presence of the coupling agent 2-ethoxy-1-ethoxycarbonyl-1,2-dihydroquinoline (EEDQ), the acid underwent amidation with commercial Phytp-Sph to yield Cer EOS, with a total yield of 19%.

**Figure 10:**
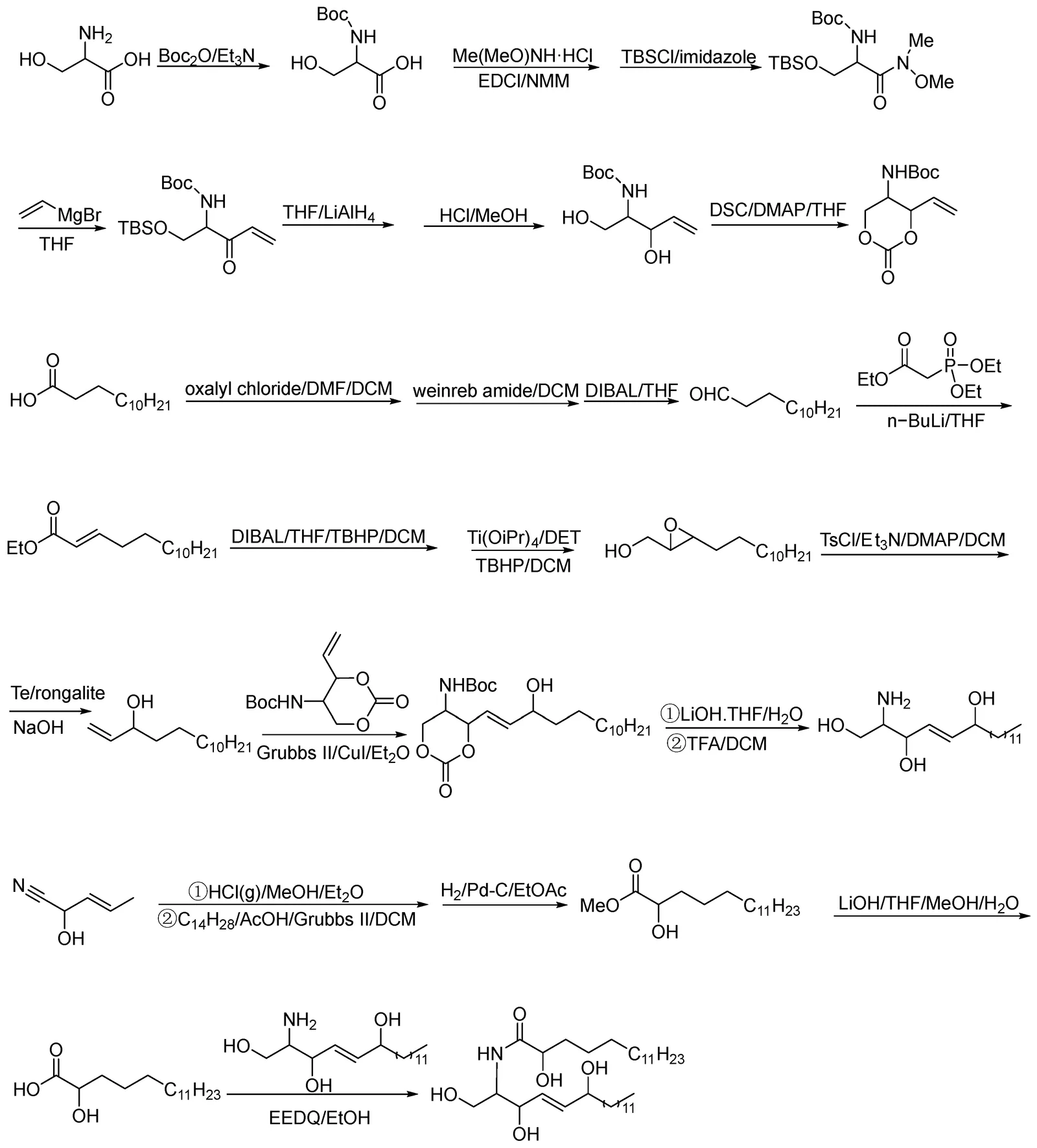
Synthesis of Cer AH [96].

Wisse et al. synthesized Cer AH using a cross-metathesis strategy (as shown in Figure 10) [96]. Serine underwent protection of the amino group, amidation of the carboxylic group, and protection of the alcohol hydroxyl group. This was followed by the formation of an unsaturated ketone with a Grignard reagent, reduction of the ketone, and deprotection of the hydroxyl group to yield an unsaturated diol. The diol then underwent condensation in the presence of N,N′-disuccinimidyl carbonate (DSC) and 4-dimethylaminopyridine (DMAP) to form a cyclic carbonate. Tridecanoic acid was converted to Weinreb amide using oxalyl chloride, which was then reduced to an aldehyde. This aldehyde underwent a Horner−Wadsworth−Emmons reaction to produce an unsaturated ester, which was subsequently reduced and subjected to Sharpless asymmetric epoxidation to obtain a chiral epoxide. The alcohol hydroxyl group of this compound reacted with TsCl to form a tosylate, which was then nucleophilically substituted by telluride ions to generate a telluride compound. This telluride compound, when exposed to air, readily formed an allylic alcohol. The allylic alcohol subsequently reacted with the previously synthesized cyclic carbonate through a series of reactions, including olefin cross-metathesis, saponification, and base deprotection, to produce 6-hydroxy sphingosine. Cyanohydrin underwent a Pinner reaction, olefin cross-metathesis, reduction, and saponification to generate fatty acid (FA). FA then underwent acylation coupling with the previously prepared 6-hydroxy sphingosine to yield the desired Cer AH, with a total yield of 26%. This synthesis utilized serine as the starting material to produce the Cer precursor—long-chain base (LCB)—providing a direction for future research on the total synthesis of Cer.

In the above synthesis, the yield of Cer EOS is less than that of Cer AH. A possible reason for this is that Cer EOS has a longer carbon chain in its fatty acid portion. During the synthesis, there may have been more losses of intermediates, resulting in a lower final yield of the product.

#### 3.3.2. Acylation Coupling Reaction of Fatty Acid Derivatives and Long-Chain Bases

**Figure 11:**
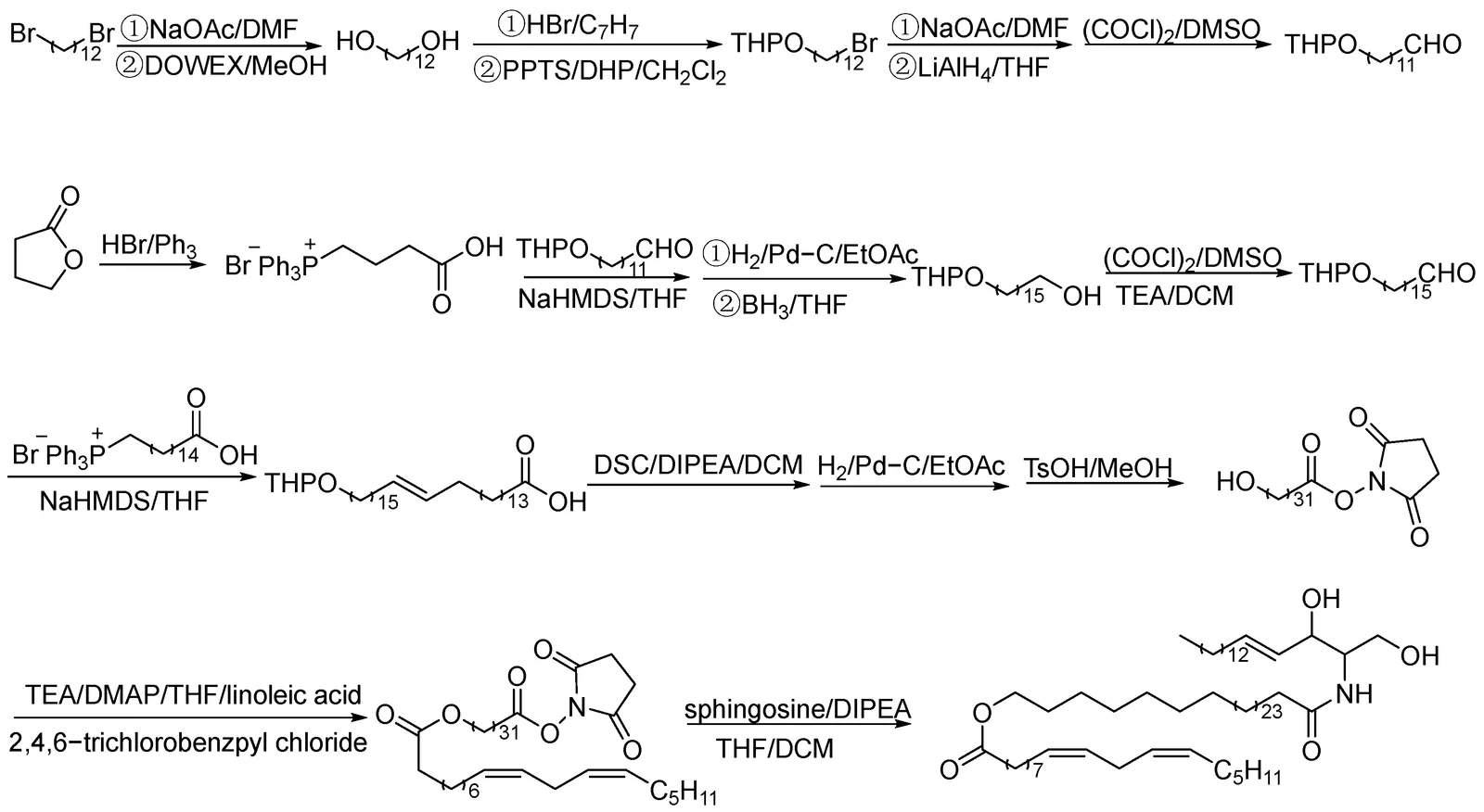
Synthesis of ultra-long-chain Cer EOS [97].

Fandrei et al. synthesized ultra-long-chain Cer EOS (as shown in Figure 11) [97]. Using 1,12-dibromododecane as the starting reactant, a series of reactions including substitution, protection of the alcohol hydroxyl group, reduction, and Swern oxidation, yielded a dodecanal with a protected hydroxyl group at the other end. This aldehyde underwent a Wittig reaction with a phosphonium salt prepared from γ-butyrolactone, followed by reduction and oxidation to produce an aldehyde with a carbon chain extended to 16 carbons. Subsequently, this aldehyde reacted with (15-carboxy pentadecyl) triphenylphosphonium bromide in a Wittig reaction to construct an ultra-long-chain unsaturated fatty acid. Next, the carboxyl group of this fatty acid was protected, followed by double bond reduction, the deprotection of the hydroxyl group, and a reaction with linoleic acid via the Yamaguchi reaction, resulting in a new ultra-long-chain fatty acid derivative. Finally, this fatty acid derivative underwent acylation coupling with commercial Phyto-Sph to yield ultra-long-chain Cer EOS, with a total yield of 2%. In this synthetic route, the formation of the fatty acid ester was achieved using the Yamaguchi reaction, which employed DMAP as a catalyst, significantly accelerating the reaction rate and improving efficiency. Although the overall yield was relatively low, the use of Wittig and Yamaguchi reactions for carbon chain elongation offers a reference for future research on the total synthesis of Cer, facilitating carbon chain extension.

**Figure 12:**
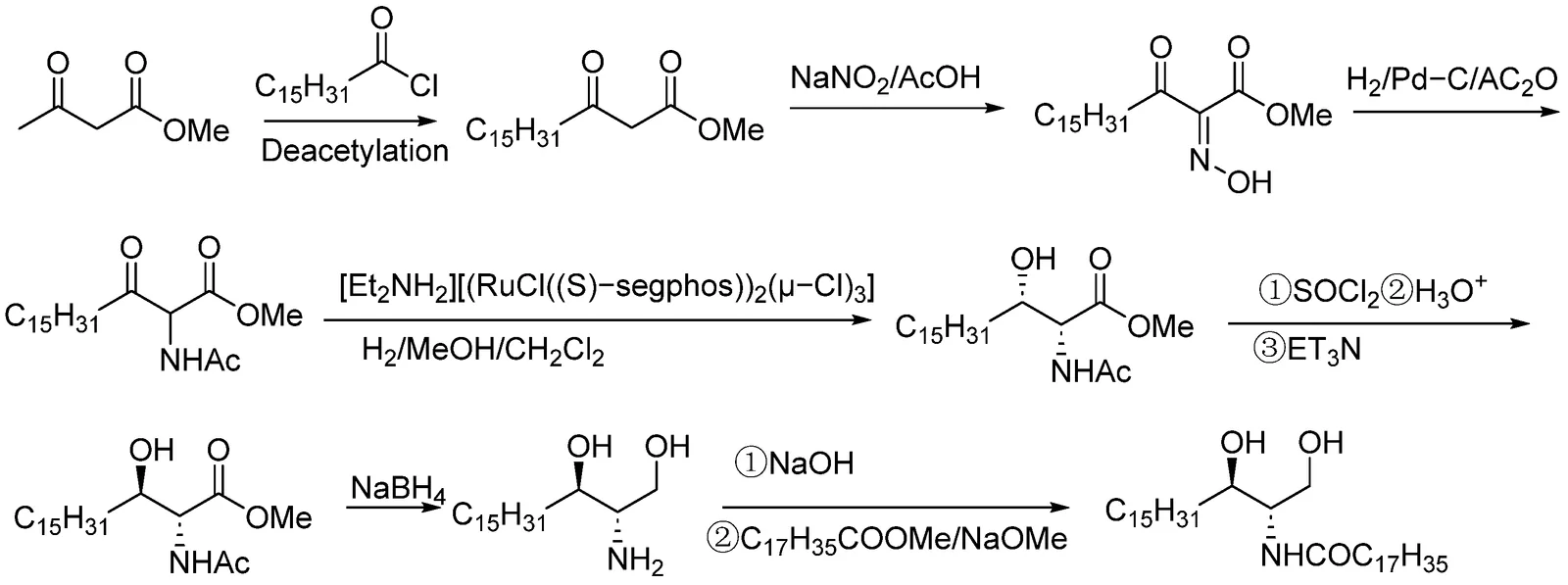
Synthesis of d-*erythro*-CER[NDS] [98].

**Figure 13:**
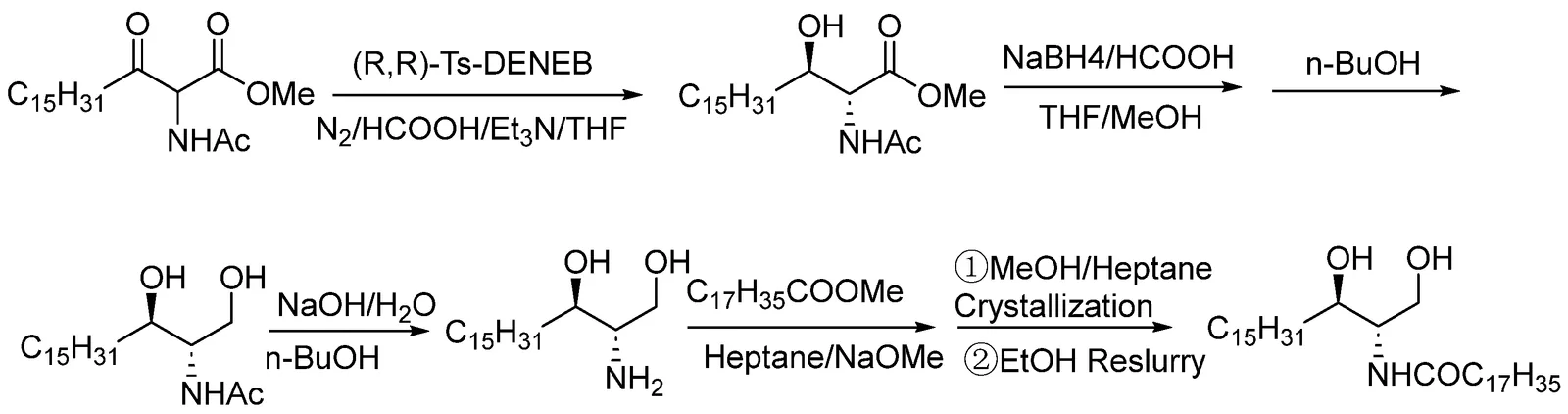
Synthesis of d-*erythro*-CER[NDS] [99].

In 2014, Ishida et al. synthesized D-erythro-CER[NDS] [98], as shown in Figure 12. The process began with deacetylation to obtain the β-keto ester, thereby extending the chain of FA. Subsequently, nitrosation and catalytic hydrogenation yielded the key intermediate methyl 2-acetamido-3-oxooctadecanoate, which could be produced in hundreds of kilograms from the initial β-keto ester, making it suitable for industrial-scale production. This intermediate was then subjected to selective hydrogenation catalyzed by Ru-segphos, followed by SOCl_2_-mediated hydroxy group configuration inversion. Finally, reduction, deacetylation, and amidation reactions were conducted to obtain D-erythro-CER[NDS]. Subsequently, Touge et al. refined Ishida’s methodology [99] (as illustrated in Figure 13). For the selective hydrogenation step, they employed their self-designed bifunctional catalyst, (R,R)-Ts-DENEB derived from sulfonylated 1,2-diphenylethylenediamine (DPEN), enabling an asymmetric transfer hydrogenation process without requiring configuration inversion. The final product, D-erythro-CER[NDS], was obtained through reduction, deacetylation, and amidation reactions. During process scale-up, the reaction was carried out in a vertical pipes-in-series reactor using 80 kg of the key intermediate, yielding 58 kg of D-erythro-CER[NDS] with a four-step yield of 47%.

#### 3.3.3. Acylation Coupling Reaction of Fatty Acid and Long-Chain Base Derivatives

**Figure 14:**
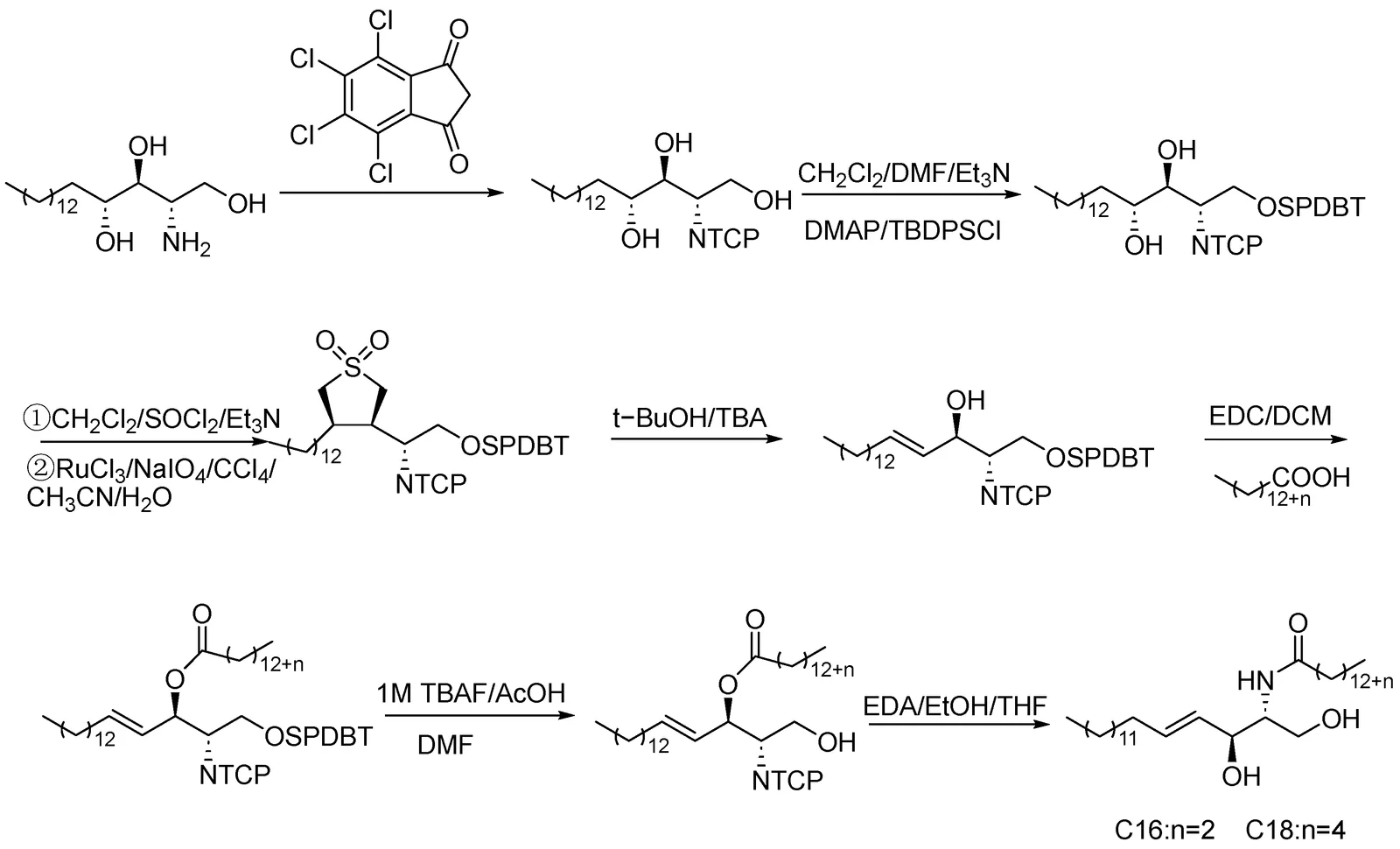
Synthesis of Cer (C16 and C18) [100].

Gorantla et al. developed an improved synthetic route to obtain Cer (as shown in Figure 14) [100]. The author adopted the method described by Roberta et al. to protect the amino and terminal hydroxyl groups of Phyto-Sph. The resulting compound reacted with SOCl_2_ to form a cyclic sulfonate [101]. They protected the amino and terminal hydroxyl groups of Phyto-Sph, and the resulting compound reacted with SOCl_2_ to form a cyclic sulfate ester. This cyclic sulfate ester was then hydrolyzed in a mixed solution of *t*-butanol and tetrabutylammonium iodide to produce the LCB derivative. This derivative first underwent a coupling reaction with fatty acid using 1-ethyl-3-(3-dimethylaminopropyl) carbodiimide (EDC), followed by the removal of the protecting group on the hydroxyl to yield the desired Cer. The total yields were 26% (C16) and 32% (C18), respectively.

**Figure 15:**
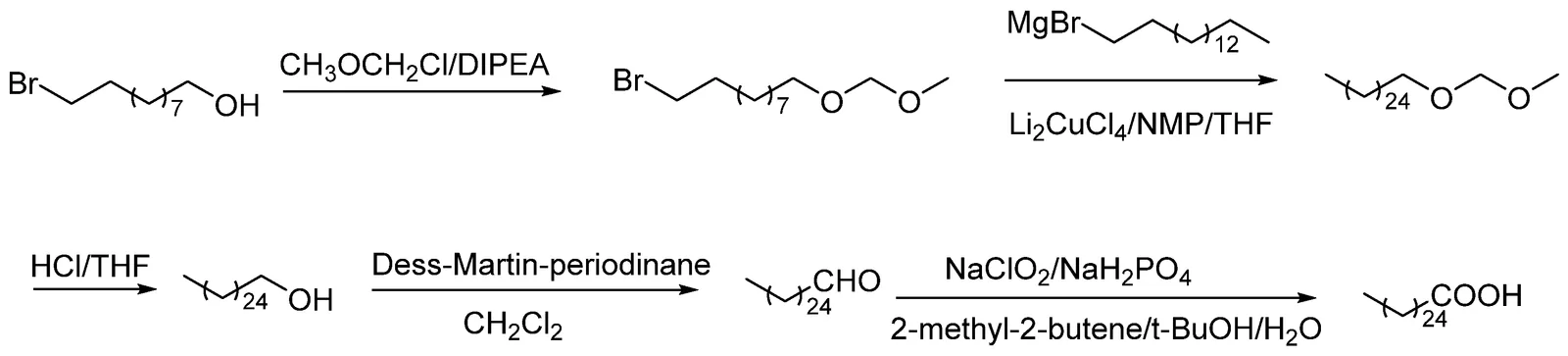
Synthesis of hexacosanoic acid [102].

**Figure 16:**
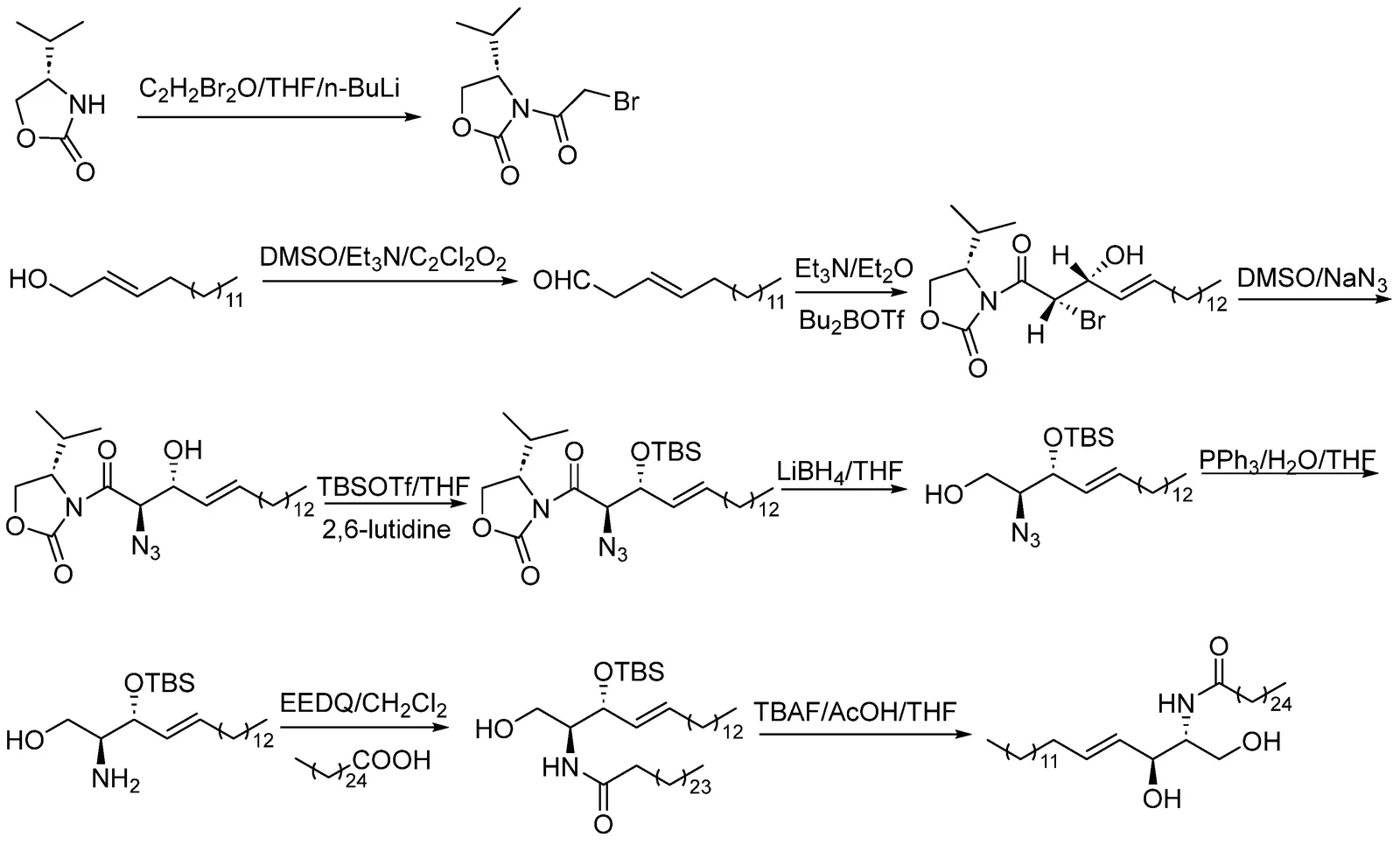
Synthesis of Cer derived from hexacosanoic acid [102].

Yamamoto et al. synthesized hexacosanoic acid and Cer derived from it [102]. The synthesis of hexacosanoic acid is shown in Figure 15. Starting from 10-bromo-1-decanol, after protecting the alcohol hydroxyl group, a Grignard coupling reaction, and evaporative hydrolysis, hexacosanol was produced. This alcohol was then oxidized using Dess-Martin periodinane and further underwent a Pinnick oxidation reaction to yield hexacosanoic acid, with a total yield of 37%. The synthesis of Cer is illustrated in Figure 16. Starting from hexacos-2-en-1-ol, a series of reactions including Swern oxidation, coupling reaction, substitution reaction, protection of the alcohol hydroxyl group, and reduction, produced an intermediate. The azido group on this intermediate was converted to an amine via Staudinger reduction. The final compound then reacted with hexacosanoic acid to form an amide. Finally, TBAF was used to remove the protecting group from the hydroxyl, yielding the final Cer compound with a total yield of 30%.

Although the two synthetic routes produced Cer from different starting materials, both processes involved the protection of the alcohol hydroxyl group. The first synthetic route used tert-butyldiphenylchlorosilane (TBDSPCl) in Figure 14, while the second employed tert-butyldimethyltrichlorosilane (TBSOTf) in Figure 16. The difference lies in the fact that using TBDSPCl requires a strong base (such as imidazole or DMAP) and a longer reaction time, and it may also need heating, resulting in higher operational costs. Additionally, due to its bulky structure, it has lower protection efficiency for sterically hindered hydroxyl groups. In contrast, TBSOTf has high reactivity and can rapidly complete silylation at low temperature. Moreover, due to its smaller steric hindrance, it can effectively protect sterically hindered hydroxyl groups. Further, in the first synthetic route, the amino group on the LCB backbone came from the starting material, whereas in the second route, it was introduced by converting an azido group through a Staudinger reaction. This method provides an alternative strategy for the introduction of amino groups in the synthesis of LCBs.

#### 3.3.4. Acylation Coupling Reaction of Fatty Acid Derivatives and Ceramide Derivatives

**Figure 17:**
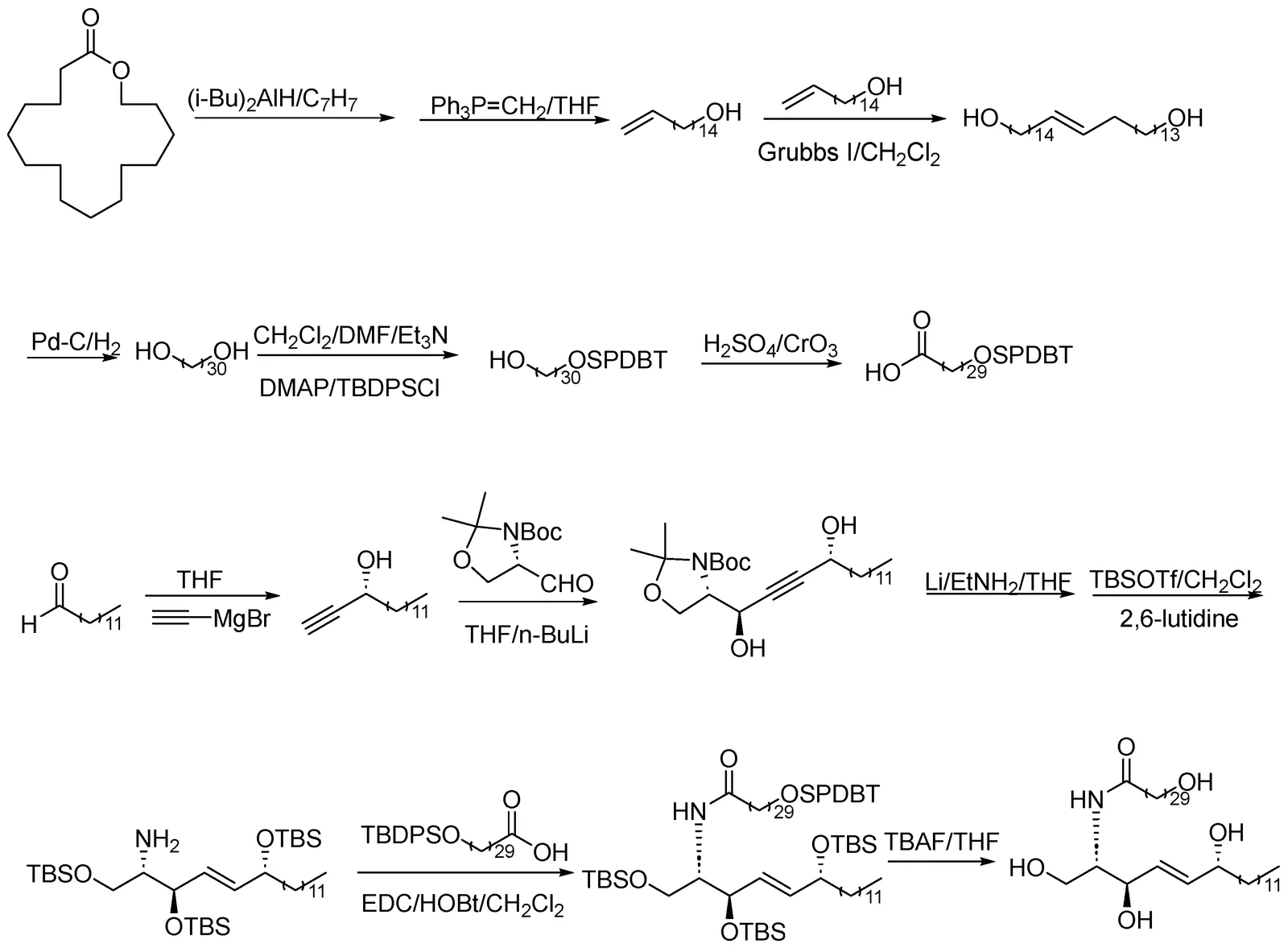
Synthesis of Cer B [103].

Mori et al. synthesized a novel ceramide with a hydroxyl group at the fatty acid chain end—Cer B (as shown in Figure 17) [103]. Using pentadecalactone as the starting material, they performed reduction and Wittig reactions to generate an unsaturated alcohol. This alcohol then underwent a cross-metathesis reaction, further reduction, deprotection of the hydroxyl group, and oxidation to produce the desired fatty acid derivative. The synthesis of the LCB derivative involved performing a Grignard reaction, addition reaction, reduction, deprotection of the amino group, and protection of the hydroxyl group on tridecanal. This LCB derivative was then coupled with the previously obtained fatty acid derivative through an acylation reaction. Finally, tetrabutylammonium fluoride (TBAF) was used to remove the protecting groups from the four hydroxyls, yielding Cer B with a total yield of 31%. This synthetic route utilized a cross-metathesis strategy to achieve carbon chain elongation and employed Gerner aldehyde as the starting material for synthesizing the LCB derivative, providing valuable insights for future research on the total synthesis of ultra-long-chain Cer.

#### 3.3.5. Synthesis of Long-Chain Bases

**Figure 18:**
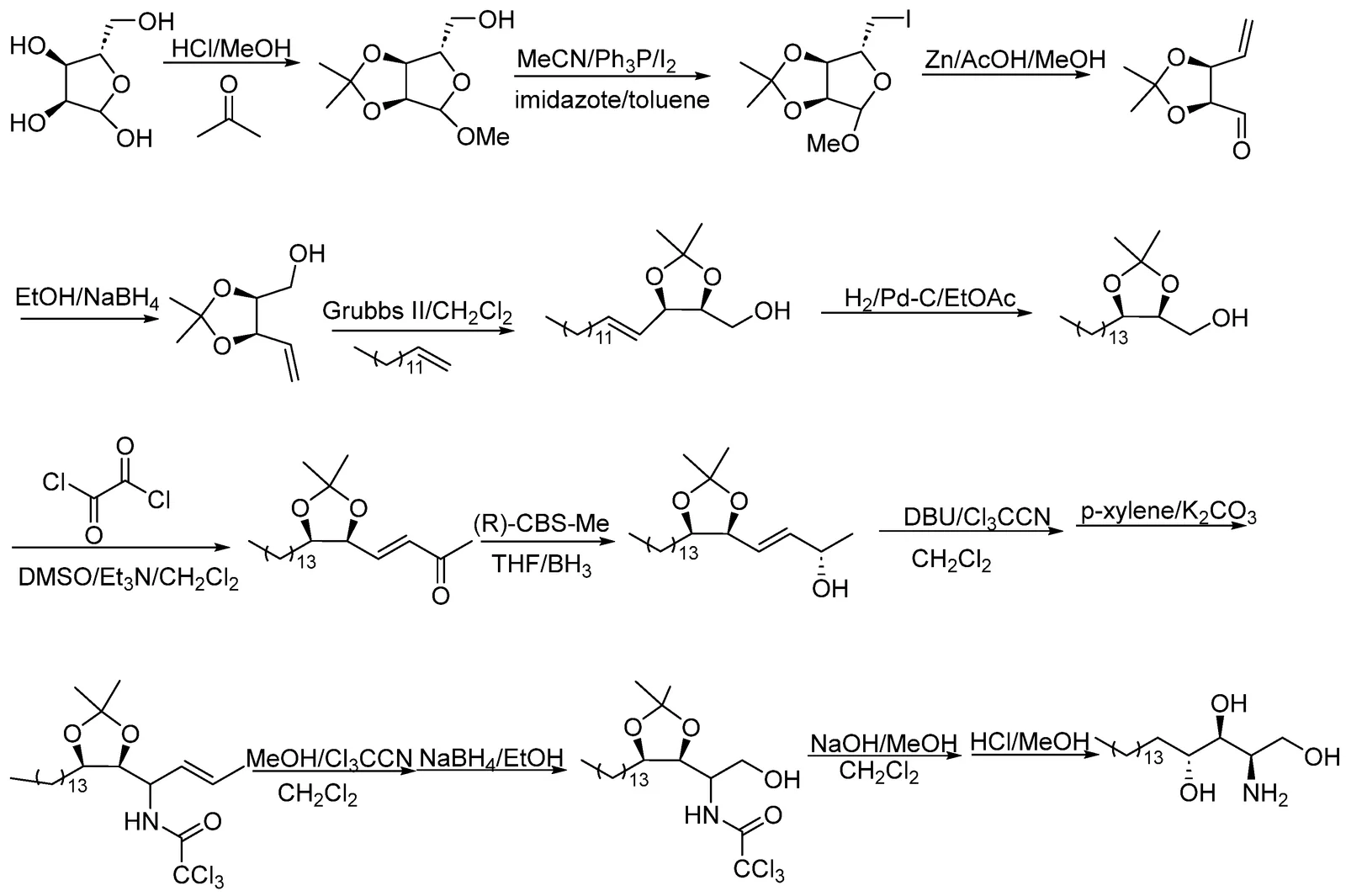
Synthesis of Phyto-Sph from D-Furanose [104].

Currently, LCBs are primarily obtained through biosynthesis, which is limited by synthesis efficiency. If LCBs can be synthesized through organic reactions, it would overcome the limitations of biosynthesis. Calder et al. synthesized Phyto-Sph using D-furanose as the starting material (as shown in Figure 18) [104]. Specifically, D-ribose underwent a mesylation reaction to produce D-furanosyl riboside. This compound then underwent a Vasella reaction, reduction, and carbene reaction to generate an alkene, achieving side-chain elongation. The resulting alkene was subjected to reduction, Swern oxidation, and Horner–Wadsworth–Emmons reaction to produce an unsaturated ketone. The unsaturated ketone was selectively reduced with (R)-Corey–Bakshi–Shibata (CBS) reagent ((R)-CBS-Me) and borane to yield (2S)-allyl alcohol. Subsequently, under standard thermal conditions (K_2_CO_3_, xylene, and 140 °C), the reaction produced allyl trichloroacetamide, which was then reduced with sodium borohydride to generate the alcohol. Following this, the trichloroacetamide group underwent alkaline hydrolysis, and the acid-participated removal of the trichloroacetamide group yielded Phyto-Sph, with a total yield of 8%.

**Figure 19:**
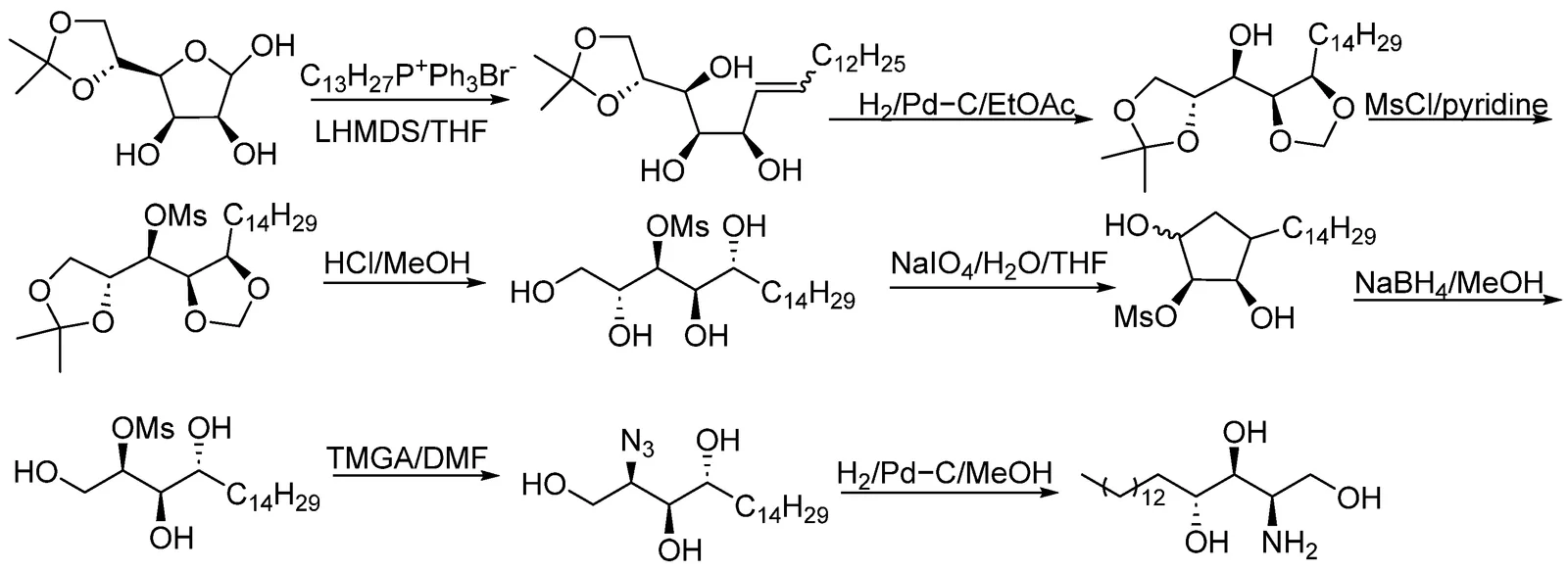
Synthesis of Phyto-Sph from a D-furanomannose derivative [105].

Chiu et al. synthesized Phyto-Sph using D-furanomannose derivative as the starting material (as shown in Figure 19) [105]. In the presence of lithium hexamethyldisilazane (LHMDS), D-furanomannose derivative underwent a Wittig reaction to produce an alkene. This alkene was then subjected to reduction and mesylation. Using HCl/MeOH as the reaction conditions, the protecting group on the aldehyde of the mesylated product was removed, yielding a tetraol derivative. Sodium periodate was used to cleave the hydroxymethyl group, resulting in a product mixture with a ratio of 1:2 (α:β) isomers. Subsequently, sodium borohydride was employed for reduction to obtain a triol compound. In this compound, the O-mesyl group was replaced with tetramethylguanidine azide (TMGA), producing an azido triol. Finally, reduction led to the formation of Phyto-Sph, with a total yield of 57%.

**Figure 20:**
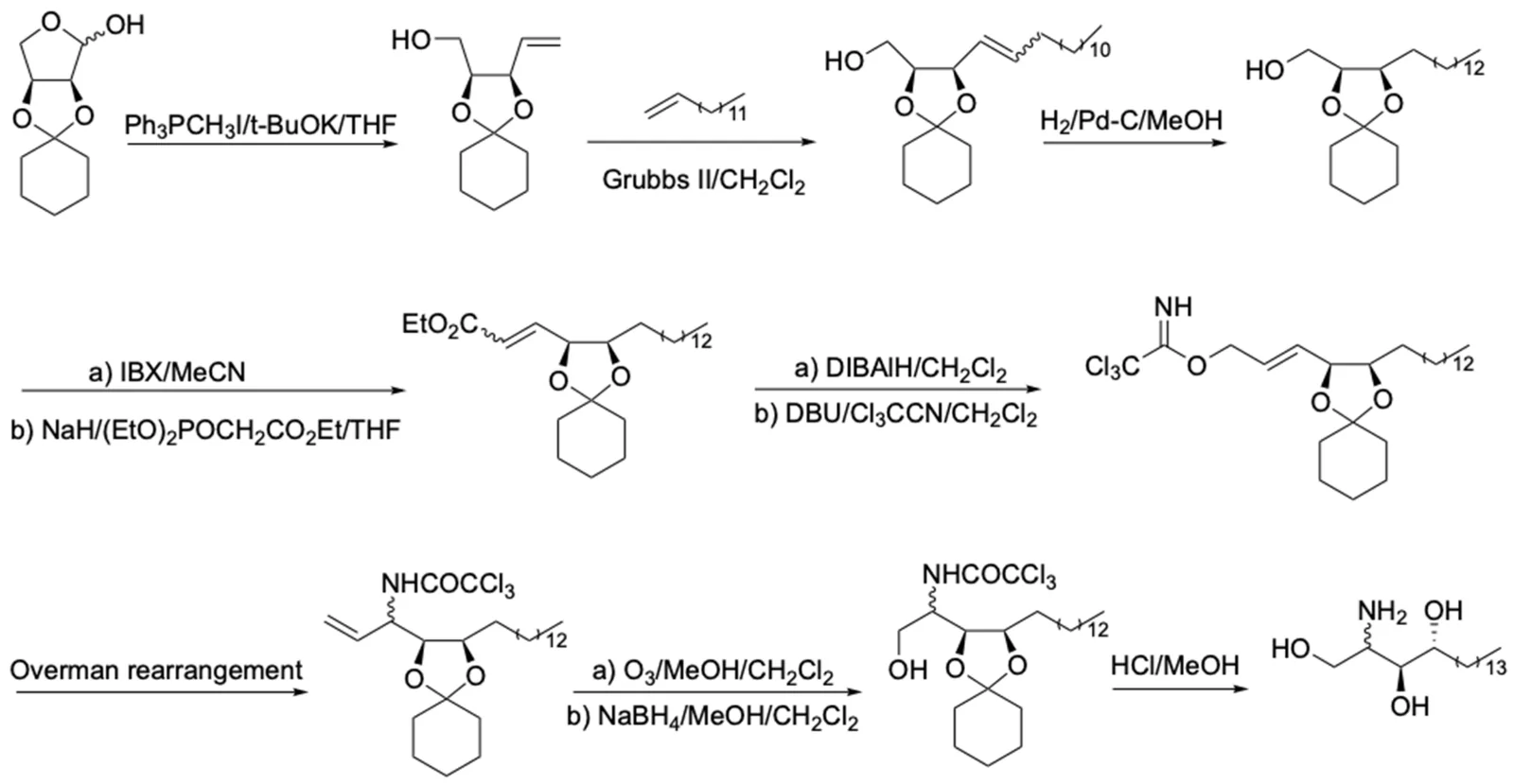
Synthesis of Phyto-Sph from D-erythrofuranose [106].

Zelinska et al. synthesized Phyto-Sph using a furanose derivative (erythrofuran scaffold) as the starting material (as shown in Figure 20) [106]. This derivative underwent a Wittig reaction, cross-metathesis reaction, and reduction to generate a saturated alcohol. The saturated alcohol was then oxidized to produce an aldehyde, which subsequently reacted with 2-(diethoxyphosphoryl)ethyl acetate to form an unsaturated ester. Following this, reduction and Overman rearrangement led to the formation of an allyl trichloroacetamide compound. The double bond in this compound underwent ozonolysis and reduction to yield a trichloroacetamide compound with a hydroxyl group. Finally, the removal of the cyclohexyl ketone and trichloroacetyl group in hydrochloric acid solution resulted in the formation of Phyto-Sph, with a total yield of 41%. This synthetic route established the LCB backbone by utilizing the erythrofuran scaffold and the Overman rearrangement of allyl alcohol. The cross-metathesis strategy was employed to extend the carbon chain, ultimately yielding the desired LCB. In future studies on the synthesis of LCBs, the introduction of amino groups can leverage the Overman rearrangement of allyl alcohol, which efficiently facilitates the migration of carbon atoms under mild conditions, significantly enhancing synthetic efficiency.

The three synthetic routes for Phyto-Sph exhibit significant variations in yield. The first synthetic route is longer, resulting in greater losses of intermediates. Additionally, all three routes involve stereoselectivity, which is highly influenced by the reaction conditions. These three synthetic routes provide valuable insights for the industrial synthesis of Phyto-Sph and lay a foundation for the realization of total synthesis processes for Phyto-Sph-type Cer.

**Figure 21:**
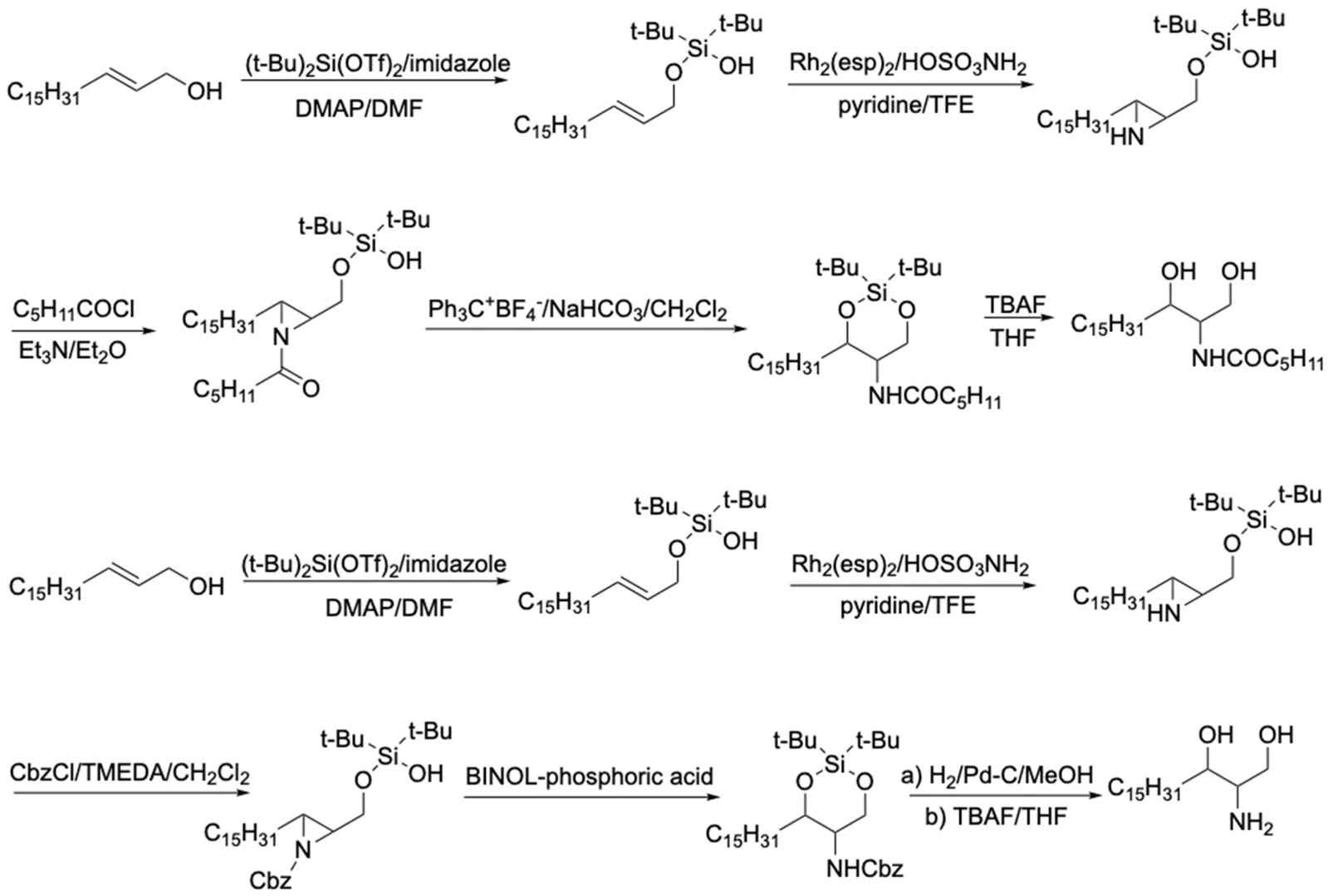
Synthesis of N-acetyl-dihydrosphingosine and dihydrosphingosine [107].

Except for Phyto-Sph, dihydrosphingosine-based Sph could also be artificially synthesized. Nagamalla et al. prepared N-acetyl-dihydrosphingosine and dihydrosphingosine using (2E)-dodecen-1-ol as the starting material through side-chain silyl alcohol to open a nitrogen-containing aziridine ring (as shown in Figure 21) [107]. Initially, the hydroxyl group on (2E)-dodecen-1-ol was protected to form a silyl alcohol. Subsequently, the double bond in the silyl alcohol underwent Kurti aziridination to produce a compound containing a nitrogen-containing aziridine ring. Finally, this compound was acetylated, cyclized, and underwent deprotection of the silyl group on the hydroxyl to yield N-acetyl-dihydrosphingosine, with a total yield of 26%. In the alternative synthetic route, the first two steps for the synthesis of dihydrosphingosine were the same as above. The compound with the nitrogen-containing aziridine ring was then protected using benzyl chloroformate (CbzCl) for the amino group, and BINOL-phosphoric acid was used to promote the cyclization reaction, followed by reduction and deprotection of the silyl group on the hydroxyl to produce dihydrosphingosine, with a total yield of 17%. This synthetic route introduced the amino group into the LCB chain through the opening of the nitrogen-containing aziridine ring. The use of silyl alcohol to facilitate the ring-opening and the removal of the silyl group produced the LCB backbone. Future research on LCB synthesis processes may adopt this method to shorten the synthetic pathway and significantly enhance reaction efficiency.

## 4. Summary and Outlook

As a group of structurally highly diverse bioactive lipids, Cer possess core structure–activity relationships determined by acyl-carbon-chain length and long-chain-base skeleton configuration. These properties directly differentiate their physiological functions and serve as key theoretical foundations connecting basic biological mechanisms, clinical disease intervention, cutaneous physiological repair, and the selection of raw-material manufacturing processes. A brief multidimensional overview indicates that within basic biological systems, Cer can cross the blood–brain barrier and act as intracellular signaling molecules to regulate cell proliferation, apoptosis, and redox homeostasis. In medical scenarios, ultra-long-chain Cer exhibit potential for tumor-therapy sensitization, antiviral effects, cardiovascular–cerebrovascular protection, and neurodegenerative-disease prevention, and plasma sphingolipid ratios can function as non-invasive biomarkers for disease screening. In skin physiological and cosmetic fields, ultra-long-chain subtypes can orderly assemble stratum-corneum lipid bilayers to realize barrier reinforcement, long-lasting moisturization, and anti-photoaging effects, whereas medium- and short-chain Cer tend to trigger epidermal oxidative damage and inflammatory responses. Distinct application scenarios impose clear directional requirements for sphingolipid carbon-chain profiles, and such structure–activity rules also deliver core evaluation criteria for screening suitable preparation technologies. Long-chain Cer exert vital functions in numerous fields. In medicine, the decline of ultra-long-chain Cer may induce certain chronic diseases and their complications [108]; in the cosmetic industry, ultra-long-chain Cer display strong molecular-association capacity, which increases stratum corneum thickness and improves skin hydration. By enhancing skin water-holding capacity, the thickened stratum corneum can more effectively resist external irritants, including ultraviolet radiation and bacteria, thereby lowering the incidence of adverse sensitive reactions such as erythema and stinging. Moreover, the thickened stratum corneum can store more natural moisturizing factors to alleviate skin dryness and desquamation and render skin softer and finer. Meanwhile, a dense, uniform stratum corneum accelerates self-repair of epidermal micro-injuries, reduces chronic-inflammation risks, and concurrently mitigates fine-line formation to maintain skin smoothness and integrity [109,110]. Apart from mainstream research directions including production-process optimization, synthetic-route development for long-chain Cer, and green synthetic transformation, the structure–activity correlation by which Cer modulate cutaneous oxidative stress and mediate antioxidant capacity constitutes another core entry point for subsequent application-oriented development, offering brand-new theoretical support for the development of functional skincare and medical repair-purpose preparations.

Systematic comparison among three mainstream acquisition strategies of Cer, namely microbial fermentation, natural biological extraction, and chemical synthesis, enables clear identification of inherent drawbacks and application limitations for each route. Microbial fermentation only yields TAPS, the precursor of Phyto-Sph, with highly restricted long-chain-base skeletons; hence, it cannot produce all the 12 types of human epidermal Cer or various functional ultra-long-chain subtypes on demand. From the perspective of biosynthetic mechanisms, natural bottlenecks exist in strain growth rates and endogenous sphingolipid metabolic pathways. Practically, fine-tuning these conditions is rather challenging, which frequently causes unstable fermentation performance, impairs TAPS productivity. High unit-product manufacturing costs consequently hinder large-scale industrial supply. Multi-parameter synergistic precise regulation over temperature, humidity, oxygen concentration, nutrients, carbon–nitrogen ratio, system pH, metal ions, and other environmental variables poses great technical difficulties. Severe batch-to-batch fluctuations in product concentration and purity readily occur during process scale-up, and production stability cannot be guaranteed, which greatly restricts the popularization of this strategy in the high-end pharmaceutical and standardized cosmetic-raw-material sectors. Natural animal- and plant-extraction processes are confronted with triple constraints regarding resource availability, production efficiency, and biosafety. Animal and plant germplasm resources rich in Cer are scarce; plant raw materials are limited by phenological cycles and geographical habitats, leading to insufficient year-round raw-material supply and failure to satisfy large-volume, stable market demands. The complete extraction workflow consists of sequential unit operations: raw-material pretreatment, multi-stage organic-solvent extraction and fine purification via silica-gel column chromatography, requiring high-equipment investment and technical barriers. For biosafety considerations, crude animal-derived Cer extracts carry potential risks of zoonotic pathogens and fail to meet raw-material-safety access criteria for oral formulations, medical dressings, and high-end cosmetics, which drastically narrows their applicable scenarios. In addition, conventional extraction consumes large quantities of toxic halogenated and alcoholic solvents such as methanol and chloroform with difficult solvent recovery, low extraction conversion rates, and poor resource utilization efficiency, so it shows no long-term advantages from environmental-protection and mass-production perspectives. By sharp contrast, chemical synthesis adopts controllable step-wise organic transformation reactions to realize directional design of long-chain-base and fatty-acid structures. It can precisely synthesize Cer with diverse carbon-chain lengths and skeleton types, perfectly matching targeted raw-material requirements derived from structure–activity relationships across various fields. Parameters of synthetic reaction systems can be rapidly iterated and optimized with short research cycles and controllable improvement of target-product conversion efficiency. Meanwhile, reaction by-products can be collected separately for resource-oriented recycling and reuse, effectively cutting overall raw-material loss and delivering prominent cost benefits. Aligned with cutting-edge trends of the green chemical industry and low-carbon manufacturing, developing low-pollution, high-atom-economy total-synthesis routes for Cer via carbon-chain elongation and acylation-coupling reactions represents a key research priority to achieve full-chain industrialization of bioactive sphingolipids. During the development on the green synthesis of ultra-long-chain Cer, simple pursuit of higher yield and lower production cost cannot serve as the sole research objective. Research should integrate practical application requirements of skin antioxidation and barrier repair, conduct targeted design of ultra-long-chain Cer and their derivatives, and achieve dual functions of barrier restoration and oxidative-stress alleviation. Furthermore, a unified efficacy-evaluation system can be constructed based on standardized in vitro stratum-corneum lipid-membrane models to quantitatively characterize Cer of distinct structures, providing referential quantitative experimental data for antioxidant cosmetic-raw-material screening and compound-formula development. Antioxidant-performance comparisons of outputs from the three mainstream preparation technologies reveal essential efficacy disparities among Cer obtained from different routes. Using a chemical-synthesis-based strategy enables the precise modulation of acyl-carbon-chain length as well as accurate separation and purification of target compounds, which allows directional production of high-purity ultra-long-chain Cer with stable and uniform antioxidant and barrier-repairing properties suitable for standardized skincare-formula development. Microbial fermentation systems merely produce the single precursor TAPS, and massive short-chain-ceramide by-products are readily generated in subsequent acylation-modification steps, which substantially weaken antioxidant activity and fail to satisfy specifications for high-activity antioxidant raw materials. Natural animal–plant extracts contain random mixtures of long- and short-chain Cer with non-adjustable component ratios and remarkable batch-to-batch antioxidant-activity fluctuations, making stable mass production of antioxidant skincare products and medical dressings impossible. Consequently, the directional preparation of ultra-long-chain Cer via chemical-synthesis routes constitutes the optimal technical approach to satisfy application demands for skin antioxidation. Future pathways for acquisition of Cer should focus on green chemical synthesis to develop more efficient and environmentally friendly processes, thereby reducing production costs and promoting broader applications in skincare.

The synthetic routes described in this review utilize the advantages of organic reactions such as the Wittig reaction, Yamaguchi reaction, and cross-metathesis of alkenes to extend ceramide carbon chains, providing research insights for future synthesis of ultra-long-chain Cer. Due to the high costs of long-chain bases (LCBs) in the market, alternative synthesis of LCBs can be achieved using furanose, furanose derivatives, and serine as starting materials to lower costs and facilitate total synthesis processes for Cer. Chiu et al. synthesized Phyto-Sph from 2,3:5,6-di-O-isopropylidene-D-mannofuranose as the starting material, achieving a total yield of 57% [105]. Based on the prices listed in Table 3, a rough calculation shows that the cost of synthesizing 1 kg of Phyto-Sph by Chiu et al. was approximately $2058 (considering only the raw material 2,3:5,6-di-O-isopropylidene-D-mannofuranose, excluding the costs of other reagents). Compared with the direct utilization of commercial LCB, the statistical data in Table 3 further demonstrates that using furanose derivatives as starting materials for LCB synthesis can more effectively reduce costs.

The method of introducing amino groups, a core component of LCBs, significantly impacts the efficiency and overall yield of ceramide synthesis. Strategies such as Staudinger reactions with azides, Overman rearrangements of allyl alcohols, and nitrogen-containing aziridine ring openings can be employed to introduce amino groups, thus shortening synthesis routes and improving reaction efficiency. In the total synthesis processes of Cer, protecting hydroxyl groups is also crucial. Protecting agents such as DHP, TBSOTf, and TBDSOCl can be used during synthesis to take advantage of different suitable environments of protecting agents, improving the strictness of reaction conditions and enhancing selectivity. Current protecting reagents generally suffer from relatively high toxicity. Future research should continuously develop novel hydroxyl-protecting reagents featuring low toxicity, facile deprotection, and high stereoselectivity. Based on structure–activity relationship, tailor-made Cer suitable for chronic-disease intervention and skin antioxidant repair scenarios should be precisely designed. Future research should continue to develop better hydroxyl-protecting agents to indirectly improve ceramide yields.

The selection of preparation methods not only determines the production efficiency of Cer but also directly influences resource utilization efficiency and environmental emission levels during the manufacturing process. Each of the three preparation methods has its advantages and disadvantages: the microbial fermentation method generates substantial waste products, imposes a high load on organic wastewater treatment systems, results in relatively high carbon emissions, and poses challenges in resource recovery; the plant extraction method requires large quantities of organic solvents, yields a lower extraction rate of Cer and necessitates significantly more organic reagents than chemical synthesis to produce equivalent quantities of Cer. While the chemical synthesis method produces only minimal amounts of waste streams, its pollutants are well-defined and amenable to centralized harmless treatment. Additionally, green catalytic and clean synthesis techniques can minimize toxic by-products, ensure high raw material utilization efficiency, avoid depletion of natural ecological resources, and offer strong production controllability, making them the preferred method for the production of Cer in alignment with green, low-carbon, and sustainable development principles. We outline multiple synthetic pathways for Cer. From a cost perspective and considering product feasibility, the two precursors FA and LCB required for Cer preparation should be synthesized de novo rather than purchased from the market at high prices (Table 3).

Besides research directions including production-process optimization, synthetic-route development for long-chain Cer, and green synthetic transformation, the structure–activity relationship serves as the underlying logic bridging molecular structure, physiological functions, and industrial-scale preparation, and acyl carbon-chain length dominates the divergence of dual biological activities of Cer. Medium- and short-chain Cer, such as C16 and C18, tend to activate inflammatory pathways, participating in pathological processes including tumor proliferation, vascular lipotoxic injury, and skin photoaging; in contrast, ultra-long-chain Cer (C22–C24) inhibit oxidative reactions, exerting protective effects in adjuvant tumor therapy, respiratory viral infection intervention, prognostic modulation of heart failure, and Alzheimer’s disease risk prevention. For the applications of Cer in diverse fields, the structure–activity relationship can also act as a critical entry point for subsequent applied research. For instance, the acyl-chain length of Cer directly determines the compactness of stratum-corneum lipids and further influences the skin’s capacity to resist oxidative damage. Ultra-long-chain Cer are capable of forming ordered and continuous lamellar lipid bilayers; such a dense lipid matrix effectively blocks the penetration of various exogenous oxidative-stress factors into the epidermis, reduces intracellular ROS accumulation, and alleviates barrier damage triggered by lipid peroxidation.

In the future optimization of chemical-synthesis systems for Cer, the core principle of green chemistry should be implemented throughout the whole preparation process, giving consideration to synthesis efficiency, economic feasibility, and environmental benignity. Conventional total-synthesis processes for Cer are plagued by cumbersome steps, high-toxicity protecting-group reagents, large organic-solvent consumption, low atom economy, and complex by-product profiles, making them incompatible with the demands of low-carbon sustainable industrialization. The iterative upgrading of subsequent synthetic processes should focus on multi-dimensional improvements: green substitution of raw materials, simplification of reaction routes, optimization of catalytic systems, and function-oriented targeted synthesis. For raw-material selection, cheap and readily available basic substrates such as furanose derivatives and L-serine can replace expensive commercial long-chain bases to cut production costs at the source and get rid of reliance on natural resources. For reaction design, highly selective organic reactions including protecting-group-free cascade synthesis, olefin cross-metathesis, and asymmetric catalysis can be employed to shorten synthetic sequences, reduce losses from intermediate isolation, and avoid frequent application of highly toxic reagents. Meanwhile, halogenated hydrocarbons and strongly polar toxic solvents should be progressively substituted, and mild catalytic as well as recyclable reaction systems are encouraged to minimize three-waste discharge. On the premise of biosafety, skeletal modification can be adopted to develop multifunctional ceramide derivatives for diversified application requirements in pharmaceuticals and functional skincare. Overall, when planning ceramide synthesis routes, we should adhere to the principles of green chemistry, striving to minimize the use of harmful solvents and reagents, thus reducing environmental impact and potential risks in various applications. Through these efforts, we can not only advance ceramide synthetic chemistry but also contribute to environmental protection initiatives.

## Figures and Tables

**Table 1:** Classification of Cer in the human skin.

	Non-Hydroxy Fatty Acid [N] 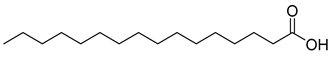	α-Hydroxy Fatty Acid [A] 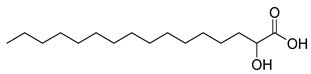	Ester-Linked ω-Hydroxy Fatty Acid [EO] 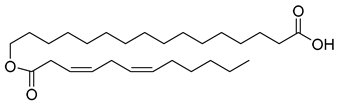
Dihydrosphingosine [dS] 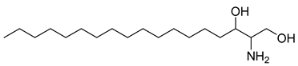	[NdS] 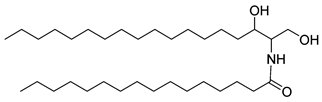	[AdS] 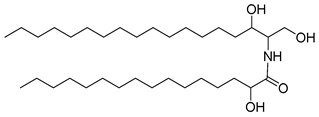	[EOdS] 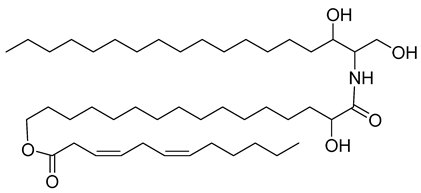
sphingosine [S] 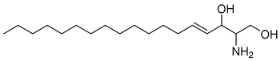	[NS] 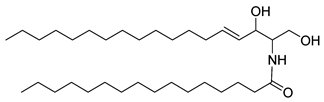	[AS] 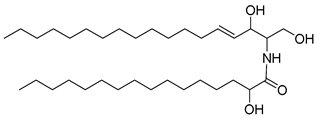	[EOS] 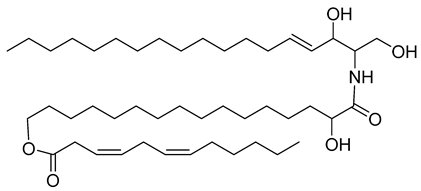
Phytosphingosine [P] 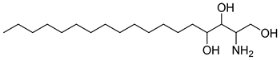	[NP] 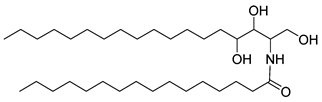	[AP] 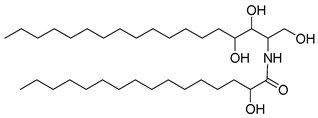	[EOP] 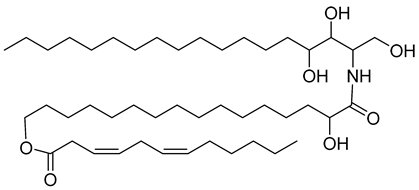
6-Hydroxysphingosine [H] 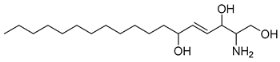	[NH] 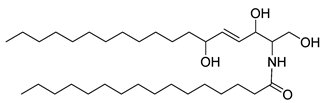	[AH] 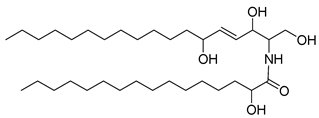	[EOH] 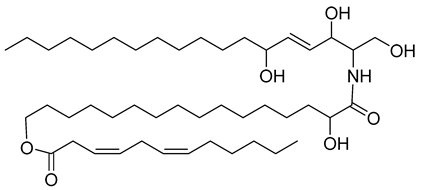

**Table 2:** Percentage of lipids extracted using SFE.

Group	SFE Conditions				Total Substances Extraction Rate (%)		Cer Extraction Rate (%)	
	T (°C)	P (atm)	VCO_2_ (mL)	% Modifier	SLS	LLS	SLS	LLS
1	60	160	350	10.0% ethanol	1.2	1.2	0.25	0.19
2	60	160	250	10.0% methanol	1.3	1.1	0.30	0.24
3	40	200	250	7.5% methanol	1.0	1.1	0.23	0.20
4	40	160	200	5.0% methanol	0.5	0.7	0.23	0.14

**Table 3:** Comparison of prices for common LCB and furanose derivatives.

Reagent	Specification	Price * ($)
D-sphingosine	250 mg	168
D-erythrodihyroseiaminol	250 mg	1362
Phyto-Sph	100 mg	679
2,3:5,6-di-O-isopropylidene-D-mannofuranose	5 g	416

* The price reference source is the official website of Shanghai Macklin Biochemical Technology Co., Ltd.

## Data Availability

No new data were created or analyzed in this study. Data sharing is not applicable to this article.
